# B cells infected with Type 2 Epstein-Barr virus (EBV) have increased NFATc1/NFATc2 activity and enhanced lytic gene expression in comparison to Type 1 EBV infection

**DOI:** 10.1371/journal.ppat.1008365

**Published:** 2020-02-14

**Authors:** James C. Romero-Masters, Shane M. Huebner, Makoto Ohashi, Jillian A. Bristol, Bayleigh E. Benner, Elizabeth A. Barlow, Gail L. Turk, Scott E. Nelson, Dana C. Baiu, Nicholas Van Sciver, Erik A. Ranheim, Jenny Gumperz, Nathan M. Sherer, Paul J. Farrell, Eric C. Johannsen, Shannon C. Kenney

**Affiliations:** 1 Department of Oncology, School of Medicine and Public Health, University of Wisconsin-Madison, Madison, Wisconsin, United States of America; 2 Department of Medical Microbiology and Immunology, School of Medicine and Public Health, University of Wisconsin-Madison, Madison, Wisconsin, United States of America; 3 Department of Pathology and Laboratory Medicine, School of Medicine and Public Health, University of Wisconsin-Madison, Madison, Wisconsin, United States of America; 4 Section of Virology, Imperial College Faculty of Medicine, Norfolk Place, London, United Kingdom; 5 Department of Medicine, School of Medicine and Public Health, University of Wisconsin-Madison, Madison, Wisconsin, United States of America; University of Utah, UNITED STATES

## Abstract

Humans are infected with two distinct strains (Type 1 (T1) and Type 2 (T2)) of Epstein-Barr virus (EBV) that differ substantially in their EBNA2 and EBNA 3A/B/C latency genes and the ability to transform B cells *in vitro*. While most T1 EBV strains contain the “prototype” form of the BZLF1 immediate-early promoter (“Zp-P”), all T2 strains contain the “Zp-V3” variant, which contains an NFAT binding motif and is activated much more strongly by B-cell receptor signalling. Whether B cells infected with T2 EBV are more lytic than cells infected with T1 EBV is unknown. Here we show that B cells infected with T2 EBV strains (AG876 and BL5) have much more lytic protein expression compared to B cells infected with T1 EBV strains (M81, Akata, and Mutu) in both a cord blood-humanized (CBH) mouse model and EBV-transformed lymphoblastoid cell lines (LCLs). Although T2 LCLs grow more slowly than T1 LCLs, both EBV types induce B-cell lymphomas in CBH mice. T1 EBV strains (M81 and Akata) containing Zp-V3 are less lytic than T2 EBV strains, suggesting that Zp-V3 is not sufficient to confer a lytic phenotype. Instead, we find that T2 LCLs express much higher levels of activated NFATc1 and NFATc2, and that cyclosporine (an NFAT inhibitor) and knockdown of NFATc2 attenuate constitutive lytic infection in T2 LCLs. Both NFATc1 and NFATc2 induce lytic EBV gene expression when combined with activated CAMKIV (which is activated by calcium signaling and activates MEF2D) in Burkitt Akata cells. Together, these results suggest that B cells infected with T2 EBV are more lytic due to increased activity of the cellular NFATc1/c2 transcription factors in addition to the universal presence of the Zp-V3 form of BZLF1 promoter.

## Author summary

EBV causes various human B cell lymphomas and infects B cells in latent or lytic forms. The two different EBV strains (Type 1 (T1) and Type 2 (T2)) are divergent in their EBNA2 and EBNA3A/B/C latency genes, and T1 EBV is more transforming than T2 EBV *in vitro*. Interestingly, all T2 strains contain the Zp-V3 form of the BZLF1 immediate-early promoter (which regulates lytic viral reactivation) while most T1 strains contain the Zp-P form; the Zp-V3 variant is more responsive to BCR stimulation due to the presence of an NFAT binding site. Here we show that T2 and T1 EBV both induce lymphomas in cord blood-humanized mice, but T2 EBV infected mice have increased lytic infection. Using *in vitro* generated LCLs, we find that both total and activated NFATc1 and NFATc2 are elevated in T2 EBV-infected LCLs compared to T1 LCLs, and demonstrate that enhanced NFAT activity is required for the lytic phenotype in T2 LCLs. Knockdown of BZLF1 gene expression decreases the growth rate of T2 LCLs. These results suggest that an increased ability to enter lytic infection may partially compensate for the decreased transforming phenotype of T2 EBV infection.

## Introduction

Epstein-Barr virus (EBV) is a herpes virus that infects most of the world’s population and causes infectious mononucleosis. EBV infection also contributes to a variety of different human malignancies, including B-cell lymphomas, T-cell lymphomas, nasopharyngeal carcinoma and gastric carcinoma. EBV establishes long-term latency in the memory B-cell compartment. During latency, the virus is maintained as a nuclear episome, only a small number of viral genes are expressed, and no progeny virus is produced [1]. EBV infection of primary B cells *in vitro* is sufficient to transform these cells into long-term lymphoblastoid cell lines (LCLs) that proliferate indefinitely and form tumors when injected into immune deficient mice. The major EBV oncoproteins (EBNA2 and LMP1) are expressed during latent infection, and human EBV-positive tumors are composed largely of latently-infected cells. Thus, latent EBV infection is required for the establishment of EBV-induced tumors. EBNA2 (which mimics constitutively active Notch signaling), LMP1 (which mimics CD40 signaling), and EBNA3C (which turns off p16 expression and prevents plasma cell differentiation) are each essential for EBV transformation of B cells *in vitro*. Nevertheless, EBV-infected lymphomas occurring in immunocompetent patients often do not express LMP1 and/or EBNA2 and EBNA3C [2,3]. Thus, the ability of EBV to transform B cells into long-term LCLs *in vitro* may not adequately model certain aspects of EBV-associated B-cell lymphomas in humans. We have recently established a humanized mouse model that better models many aspects of the human disease, including a role for lytic infection [4], and the development of EBV-induced lymphomas in the absence of LMP1 or EBNA3C [5,6].

Lytic infection, in which progeny virus is produced, occurs specifically in antigen-stimulated B cells, plasma cells and oropharyngeal epithelial cells [7–9]. Lytic EBV infection is required for horizontal transfer of the virus from cell-to-cell and host-to-host, and when confined to a subset of tumor cells may contribute to EBV-positive tumors by increasing the total number of latently-infected cells, and/or by inducing paracrine factors that support the growth of latently-infected tumor cells [10–13]. Enhanced lytic viral infection occurs early in the development of EBV-positive NPC [14], and immunosuppressed organ transplant recipients (who are at high risk for EBV-induced lymphoproliferative disease) have increased lytic, as well as latent, EBV infection [15]. The ability of malaria co-infection in children to promote the development of EBV-positive Burkitt lymphoma is also associated with greatly increased lytic EBV infection [16–18].

Lytic EBV infection in B cells is mediated by cellular factors that activate expression of the EBV BZLF1 (Z) immediate-early (IE) gene, which encodes a transcription factor that cooperates with the EBV BRLF1 (R) IE protein to induce expression of early lytic viral promoters [19]. B-cell receptor (BCR) stimulation induces lytic EBV reactivation very strongly in many EBV-infected Burkitt lines (BLs), and we recently showed that BCR-mediated lytic reactivation in EBV+ BLs requires the cellular NFATc1 transcription factor [20]. In addition, we found that viral strains containing the Zp-V3 form of the BZLF1 promoter (Zp), which contains an NFAT binding motif, are activated more strongly by BCR stimulation than strains containing the prototype Zp form (Zp-P) [20].

Humans are infected with two different types of EBV (Type 1 (T1) and Type 2 (T2)), but relatively little is known about T2 EBV. EBV strains are classified as T1 or T2 according to differences in their EBNA2 and EBNA 3A/B/C latency gene sequences, while there is very little variation in most other EBV genes [21,22]. However, there are consistent differences in some of the EBV lytic genes including gp350 and gp42 [23,24]. T1 EBV infection is thought to be much more common than T2 EBV infection in western countries, and most EBV experimentation has been performed using T1 EBV strains [21,22]. T2 EBV infection in the blood is most commonly found in sub-Saharan Africa and New Guinea, where it is present in ~25% of individuals [21,22,25]. Nevertheless, T2 EBV (usually along with T1 EBV) was recently shown to be present in ~50% of 53 saliva specimens from healthy UK university students that specifically had high levels of EBV in saliva [23,24]. Humans can be simultaneously infected with both EBV types (particularly immunosuppressed patients) [26,27], and very occasional recombination between T1 and T2 strains has been found in sequenced genomes [23].

The major previously reported phenotypic difference between T1 and T2 EBV infection *in vitro* is that T1 EBV transforms B cells more efficiently than T2 EBV; this difference was first reported to be due to a single amino acid difference (S442D) between the EBNA2 proteins that allows the T1 EBNA2 to bind to EICE sites and more efficiently activate several cellular genes and the LMP1 promoter [28–32]. A further mechanism involving an additional binding site in type 2 EBNA2 for the cell repressor BS69 has also been found [31]. In addition, T2 EBV was also recently reported to infect T cells more efficiently [33–35]. Since LMP1 is critical for B-cell transformation *in vitro*, reduced LMP1 expression in T2 EBV–infected B cells likely contributes to T2 EBV’s transformation defect *in vitro*. Nevertheless, since we previously showed that an LMP1-deleted EBV mutant can induce lymphomas in a cord blood-humanized mouse model [5], and another group recently found that a T2 EBV strain (LCL-10) produces lymphomas in humanized mice with a similar efficiency as a T1 EBV strain (B95.8) [33], it is not clear that T2 EBV is less transforming than T1 EBV under more physiologic circumstances.

Although differences between T1 versus T2 EBV genomes are most prominent in the EBNA2 and EBNA3A/B/C latency genes, analysis of multiple different EBV genome sequences has revealed that all T2 EBV strains contain the Zp-V3 form of the Zp, whereas most T1 strains have the “prototype” form, Zp-P [23,24]. Thus, T1 and T2 EBV may have functionally significant differences in Zp activity, but the biological importance of these differences has not yet been examined. In this study, we have compared the phenotypes of LCLs infected with T1 versus T2 EBV strains, and lymphomas infected with T1 versus T2 EBV in cord blood-humanized (CBH) mice. We found that both T1 and T2 EBV cause tumors in the CBH mouse model, although consistent with previous *in vitro studies* tumors infected with type 2 EBV express less LMP1 [28]. Importantly, we show that T2 EBV infected B cells are much more constitutively lytic in comparison to T1 EBV infected cells, both in CBH lymphomas and *in vitro* LCLs. Using the LCL model, we have explored the mechanism(s) mediating enhanced lytic infection in B cells with T2 EBV infection. Since Akata virus-infected LCLs are highly latent, even though the T1 Akata virus has the Zp-V3 form of Zp, the presence of Zp-V3 per se does not appear to be sufficient for this constitutively lytic phenotype. We show that two different members of the NFAT transcription factor family, NFATc1 and NFATc2, have elevated activity in T2 infected LCLs and that lytic BZLF1 protein expression is dependent on NFAT activity, with NFATc2 having the stronger effect. These results reveal that a major phenotypic difference between T1 EBV- versus T2 EBV- infected B cells is increased lytic viral infection in T2 EBV-infected cells and suggest that this altered phenotype is due to not only the universal presence of the Zp-V3 form of Zp in T2 EBV strains, but also enhanced NFATc1/NFATc2 activity in B cells with T2 EBV infection.

## Results

### Type 1 and Type 2 EBV viruses both induce aggressive B-cell lymphomas *in vivo* in cord blood-humanized mice

Type 2 (T2) EBV has been previously reported to be less efficient than Type 1 (T1) EBV at transforming primary B cells *in vitro*, and this effect has been associated with a lower level of LMP1 expression in T2 EBV-infected LCLs [28,29]. However, given our recent studies showing that LMP1 is not required for EBV-induced (T1 B95.8 strain) lymphomas in CBH mice, we hypothesized that T2 EBV can form lymphomas in CBH mice as efficiently as T1 EBV, even though T2 EBV is at least partially deficient for transformation of B cells *in vitro* [30]. Furthermore, another group, using one T1 EBV strain (B95.8) and one T2 EBV strain (LCL-10) recently reported a similar ability of the T1 versus T2 EBV strains to induce lymphomas in a humanized mouse model [33].

**Fig 1:**
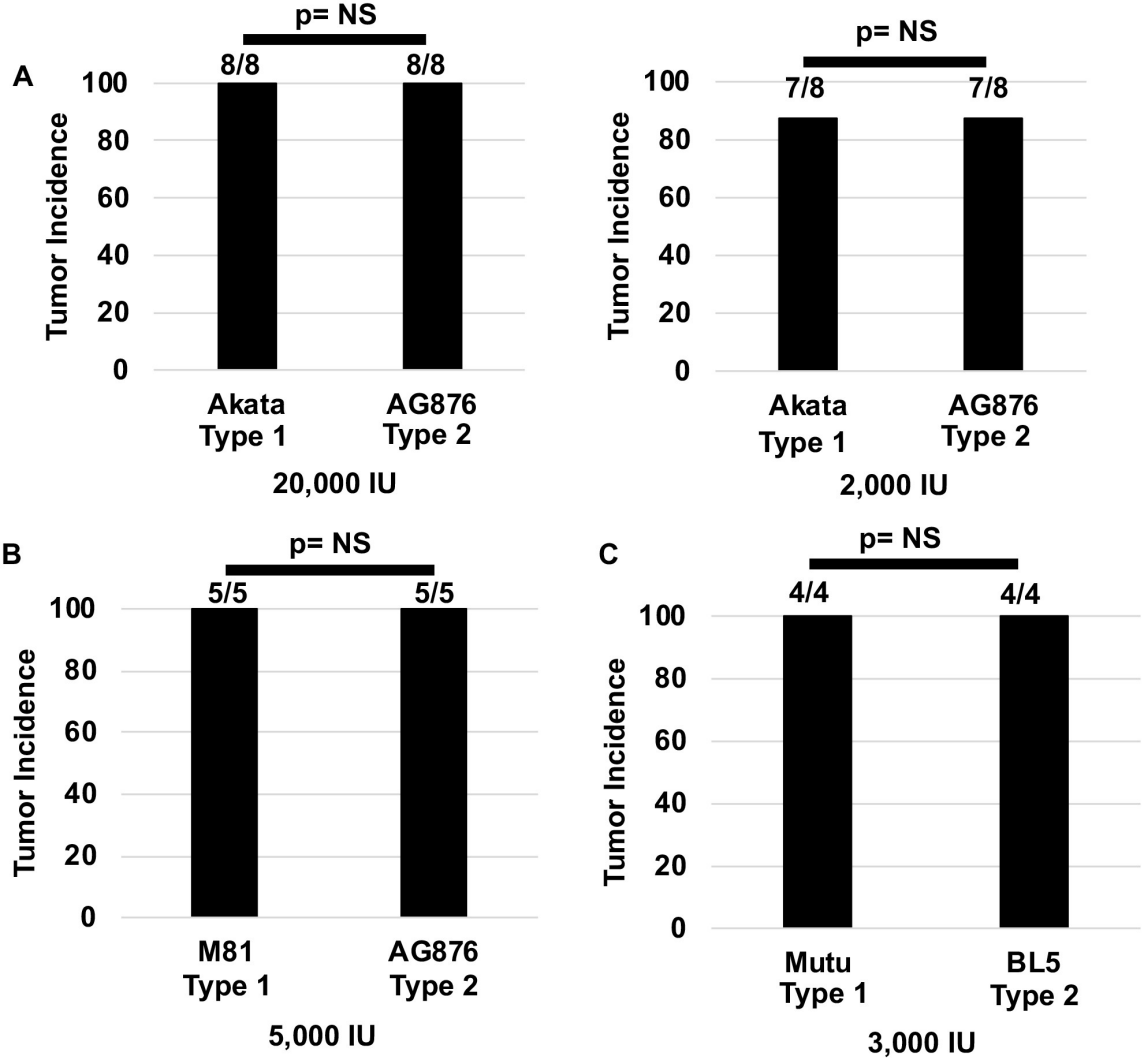
T2 EBV infected CBH mice develop tumors at a similar frequency as T1 EBV infected mice. **A)** The proportion of CBH mice that developed tumors after injection with 20,000 or 2,000 infectious units of either T1 Akata or T2 AG876 is shown. Two experiments were performed using both doses with two different donors. **B)** The proportion of CBH mice that developed tumors using 5,000 infectious units of T1 M81 or T2 AG876. **C)** The proportion of CBH mice developing tumors using 3,000 infectious units of T1 Mutu and T2 BL5 viruses. Fisher’s exact test was performed to determine if tumor frequency rates were significantly different between the different conditions.

To compare T1 versus T2 EBV phenotypes in our cord blood-humanized mouse model, we initially compared the effect of using two different doses (20,000 or 2,000 infectious units) of EBV isolated from either Akata BL cells or AG876 BL cells. Akata is a T1 EBV strain while AG876 is a T2 strain; both contain the Zp-V3 form of the Z promoter. In each experiment, cells derived from the same donor were used to compare the phenotypes of the two virus types. Following infection with each virus, animals were euthanized at day 35 and the incidence of lymphomas was determined by both gross examination of organs and by H&E and IHC analysis of FFPE tissue slides containing portions of multiple organs (including intra-abdominal omentum, lymph nodes, spleen, pancreas, gall bladder, small and large bowel, stomach, lungs, and kidney) from each animal. As shown in Fig 1A, both the Akata and AG876 viruses induced lymphomas in CBH mice, and the locations of these lymphomas were similar in animals infected with either virus (Table 1). We obsevered no obvious difference in tumor volume and time it took for tumors to develop in the CBH mouse model.

**Table 1:** The location of the various tumors generated in CBH mice used in this study.

Animal ID	Virus and Type	Dose of Virus	Location
367L	Akata (T1)	2,000 IU	Pancreas, bile duct, mesentery, lung, kidney, liver, spleen, gall bladder
368L	Akata (T1)	2,000 IU	Pancreas, mesentery, lung, kidney, liver, spleen
369L	Akata (T1)	2,000 IU	Pancreas, esophagus, diaphragm, liver, spleen, kidney, mesentery, lung
371R	Akata (T1)	2,000 IU	Pancreas, liver, kidney, mesentery
SK1478	Akata (T1)	2,000 IU	Pancreas, spleen, kidney, mesentery, lung, liver
SK1482	Akata (T1)	2,000 IU	Pancreas, liver, small intestine, mesentery, gall bladder, spleen, lung kidney
SK1484	Akata (T1)	2,000 IU	Pancreas, mesentery, kidney, liver, lung, gall bladder, spleen
SK1515	Mutu (T1)	3,000 IU	Pancreas, diaphragm, kidney, liver, mesentery
SK1519	Mutu (T1)	3,000 IU	Pancreas, lung, mesentery, muscle, liver, spleen
SK1521	Mutu (T1)	3,000 IU	Muscle, liver, mesentery, spleen
368LR	AG876 (T2)	2,000 IU	Pancreas, mesentery, lung, spleen, liver, kidney
369LR	AG876 (T2)	2,000 IU	Pancreas, mesentery, lung, spleen, subcutaneous
370L	AG876 (T2)	2,000 IU	Pancreas, mesentery, lung, spleen, kidney, bile ducts
371Φ	AG876 (T2)	2,000 IU	Pancreas, spleen, lung, liver, kidney
SK1477	AG876 (T2)	2,000 IU	Pancreas, liver, mesentery
SK1479	AG876 (T2)	2,000 IU	Pancreas, lung, liver
SK1481	AG876 (T2)	2,000 IU	Para pancreatic bile duct
SK1483	AG876 (T2)	2,000 IU	Pancreas, bile duct, kidney, liver, mesentery
SK1516	BL5 (T2)	3,000 IU	Pancreas, spleen, liver, mesentery, diaphragm, lung
SK1517	BL5 (T2)	3,000 IU	Pancreas, liver, lung, kidney, small intestine, spleen
SK1518	BL5 (T2)	3,000 IU	Pancreas, spleen, mesentery, lung, subcutaneous
SK1520	BL5 (T2)	3,000 IU	Pancreas, liver, bile duct, gall bladder, spleen, lung

We next compared the ability of the T1 M81 (Zp-V3 containing) EBV strain versus the T2 AG876 EBV strain to form lymphomas in CBH mice (using a dose of 5000 infectious units per mouse). This experiment also resulted in lymphomas occurring following infection with either T1 or T2 EBV strains (Fig 1B). To confirm that the ability of T2 EBV to induce lymphomas in CBH mice is not unique to the T2 AG876 strain, and to determine if different results would be obtained using a T1 EBV strain containing the Zp-P form of the Z promoter, we also compared the ability of another T2 EBV virus (BL5), versus another T1 virus (Zp-P containing Mutu) to form lymphomas in the CBH model (using a dose of 3000 infectious units per mouse). As shown in Fig 1C, both Mutu and BL5 viruses induced lymphomas in the majority of infected animals in the CBH model. Together, these results suggest that both T1 and T2 EBV strains can induce lymphomas in the CBH model, at least when a dose of 2000 infectious units of EBV or higher is given per animal. Since we have previously found that infectious T1 EBV doses of less than 2000 units per animal do not reproducibly induce tumors in the majority of infected animals, we did not compare the effects of lower doses of T1 versus T2 virus infection, due to the expense of this model. Thus, differences in the ability of T1 versus T2 EBV to induce lymphomas in the CBH model that only occur using very low doses of EBV cannot be totally excluded.

### Both T1 and T2 EBV induce activated diffuse large B cell lymphomas (DLBCLs) with Type III latency in CBH mice, but T1 EBV-infected tumors express more LMP1

**Fig 2:**
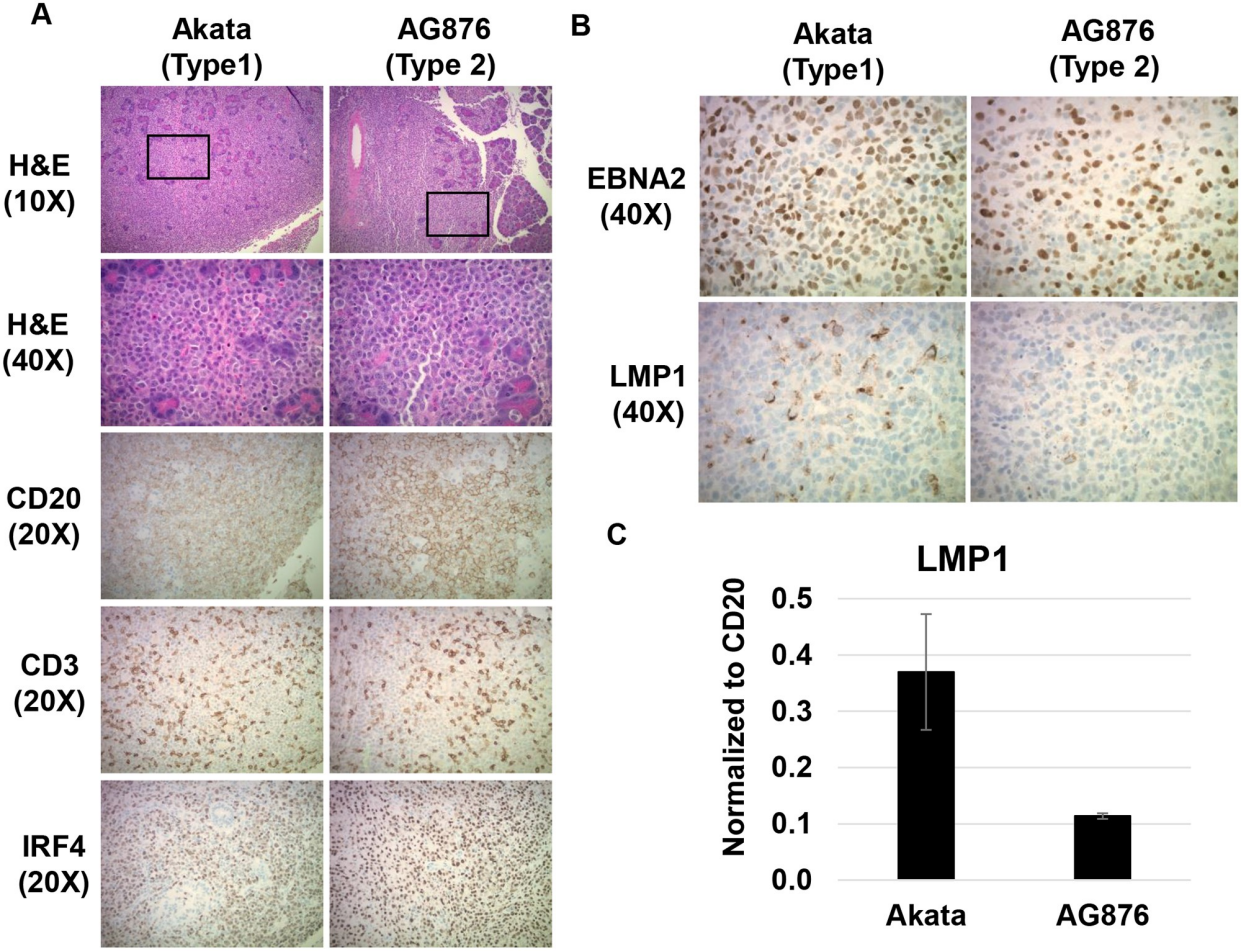
T2 EBV induces activated DLBCLs that are in Type III latency but express less LMP1 than T1 EBV-induced lymphomas. **A)** H&E staining was performed on T1 and T2 EBV-induced lymphomas, each invading the pancreas. IHC analysis was performed using antibodies against CD20 (B cell marker), CD3 (T cell marker) and IRF4 (maker of activated DLBCLs) as indicated. **B)** IHC analysis of T1- and T2-induced lymphomas using antibodies against EBNA2 (EBV latency protein) and LMP1 (EBV latency protein) as indicated. **C)** qPCR analysis of RNA isolated from T1 and T2 EBV-induced lymphomas using primers that recognize both T1 and T2 LMP1 genes; results were normalized to the level of the cellular B-cell specific CD20 transcript.

To compare the phenotypes of lymphomas induced by T1 versus T2 EBV in the CBH mouse model, tumors were stained with hematoxylin and eosin (H&E), and immunohistochemical (IHC) staining was performed with multiple antibodies to assess the cellular composition as well as viral expression pattern. Both T1 and T2 EBV-infected lymphomas were distinctly invasive in nature, and were commonly found invading the pancreas, gallbladder, and liver (Fig 2 and Table 1). Tumors induced by either the Akata or AG876 virus types were phenotypically similar to human DLBCLs on H&E stains and expressed IRF4 (a marker for the activated form of DLBCLs). T1 and T2 EBV-infected tumors also expressed similar levels of the B-cell marker CD20 and were infiltrated by a similar number of CD3-positive T cells (Fig 2A). Similar results were observed in lymphomas induced by the T1 Mutu virus and T2 BL5 virus.

To examine expression of EBV latency proteins in T1 EBV- versus T2 EBV-infected lymphomas, we performed EBNA2 and LMP1 IHC analysis of Akata virus- and AG876 virus-infected tumors. Although Akata- and AG876-infected tumors contained a similar number of EBNA2-positive cells, the number of LMP1-positive cells appeared to be decreased in AG876 virus-infected tumors relative to the Akata virus-infected tumors (Fig 2B). LMP1-expressing cells were likewise more frequent in tumors infected with T1 Mutu virus versus T2 BL5 virus. To compare the level of LMP1 expression in Akata virus- versus AG876 virus-infected cells more quantitatively, we isolated RNA from two Akata virus-infected tumors and two AG876 virus-infected tumors and performed qPCR analysis using PCR primers that can detect both T1 and T2 LMP1 transcripts [36]. T2 AG876 EBV-induced lymphomas expressed nearly 4-fold less LMP1 transcript compared to their T1 Akata virus-infected counterparts (Fig 2C), although the levels of the B-cell specific CD20 transcript were similar in each tumor type. These results indicate that both T1 and T2 EBV induce DLBCs with Type III viral latency in CBH mice, although T1 EBV infection in CBH mice *in vivo* results in higher levels of LMP1 expression, similar to what has been reported in newly EBV-infected B cells *in vitro* [28,29].

### T2 AG876 EBV-infected lymphomas have highly elevated levels of lytic viral proteins compared to T1 Akata and T1 M81 EBV-infected lymphomas

**Fig 3:**
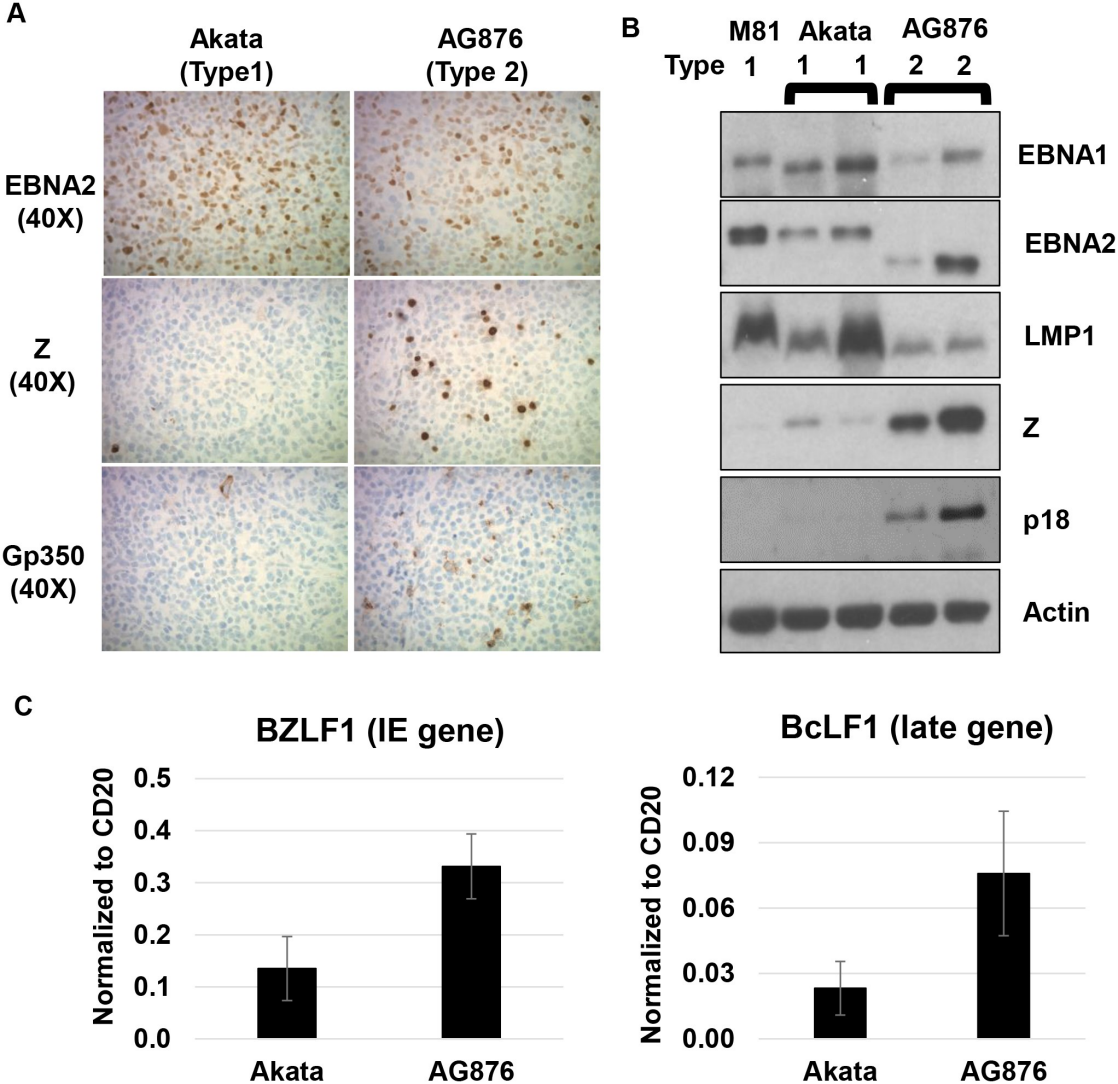
T2 EBV-infected lymphomas have elevated lytic infection compared to T1 EBV-infected lymphomas. **A)** IHC analysis using antibodies against EBNA2 (EBV latency protein), BZLF1 (Z) (immediate-early lytic protein), and gp350 (late lytic protein) was performed as indicated. **B)** Immunoblot analysis of proteins isolated from T1 and T2 lymphomas was performed using antibodies against EBNA1 (EBV latency protein), EBNA2, LMP1 (EBV latency protein), Z, p18 VCA (late lytic protein), and actin. **C)** RNA isolated from T1 and T2 EBV-induced lymphomas was subjected to qPCR analysis using primers that recognize the immediate early lytic gene BZLF1 and late lytic gene BcLF1 from both T1 and T2 EBV; results were normalized to the level of the cellular B-cell specific CD20 transcript.

We next compared the frequency of lytically-infected lymphoma cells in tumors infected with T1 versus T2 EBV. We hypothesized that lymphomas infected with T2 viruses may be more lytic than lymphomas infected with T1 viruses, since LMP1 is reported to inhibit lytic viral reactivation in B cells, and all T2 viruses have the more active form of the Z promoter (Zp-V3) [23,24,37]. To compare the level of lytic viral protein in tumors with T1 versus T2 EBV infection, we initially performed IHC studies using antibodies that detect either BZLF1 (Z), an immediate-early lytic viral protein, or gp350 (a late lytic structural viral glycoprotein). As shown in Fig 3A, AG876 EBV-infected lymphomas contained strikingly more Z-staining and gp350-staining cells on IHC analysis in comparison to Akata virus-infected tumors. Similar results were also observed when comparing the T2 BL5 strain and T1 (Zp-P containing) Mutu strain (S1 Fig). We previously showed that infection with another T1 EBV strain (B95.8) also results in highly latent EBV infection in CBH mice [5,6,38].

To confirm that tumors infected with T2 EBV have a higher level of lytic viral protein, we also assessed the amount of lytic and latent viral protein expression in T1 versus T2 virus-infected lymphomas in CBH mice by extracting protein from individual tumors infected with each virus type and performing immunoblot analyses. As shown in Fig 3B, there was no consistent difference in the amount of EBNA2 or EBNA1 expressed in T1 versus T2 virus-infected tumors (although as expected EBNA2 has a smaller molecular weight in T2 tumors). As suggested by our IHC analysis, LMP1 protein was expressed at lower levels in the T2 virus-infected tumors. However, expression of the lytic viral proteins, including the immediate-early protein Z, and the late lytic protein p18, was much higher in tumors infected with T2 EBV. We also isolated RNA from two Akata virus-infected tumors and two AG876 virus-infected tumors and performed qPCR analysis to quantitate the transcript levels of the BZLF1 IE gene and the BcLF1 late lytic gene (which encodes the major viral capsid protein). As shown in Fig 3C, T2 AG876 EBV-infected lymphomas clearly expressed more BZLF1 and BcLF1 transcripts compared to T1 Akata EBV-induced lymphomas. Together, these results suggest that the most striking phenotypic difference between T1 and T2 EBV infection of CBH mice *in vivo* is the enhanced lytic viral infection in lymphomas containing T2 EBV.

### T2 EBV primarily infects B cells (rather than T cells) in cord blood-humanized mice

**Fig 4:**
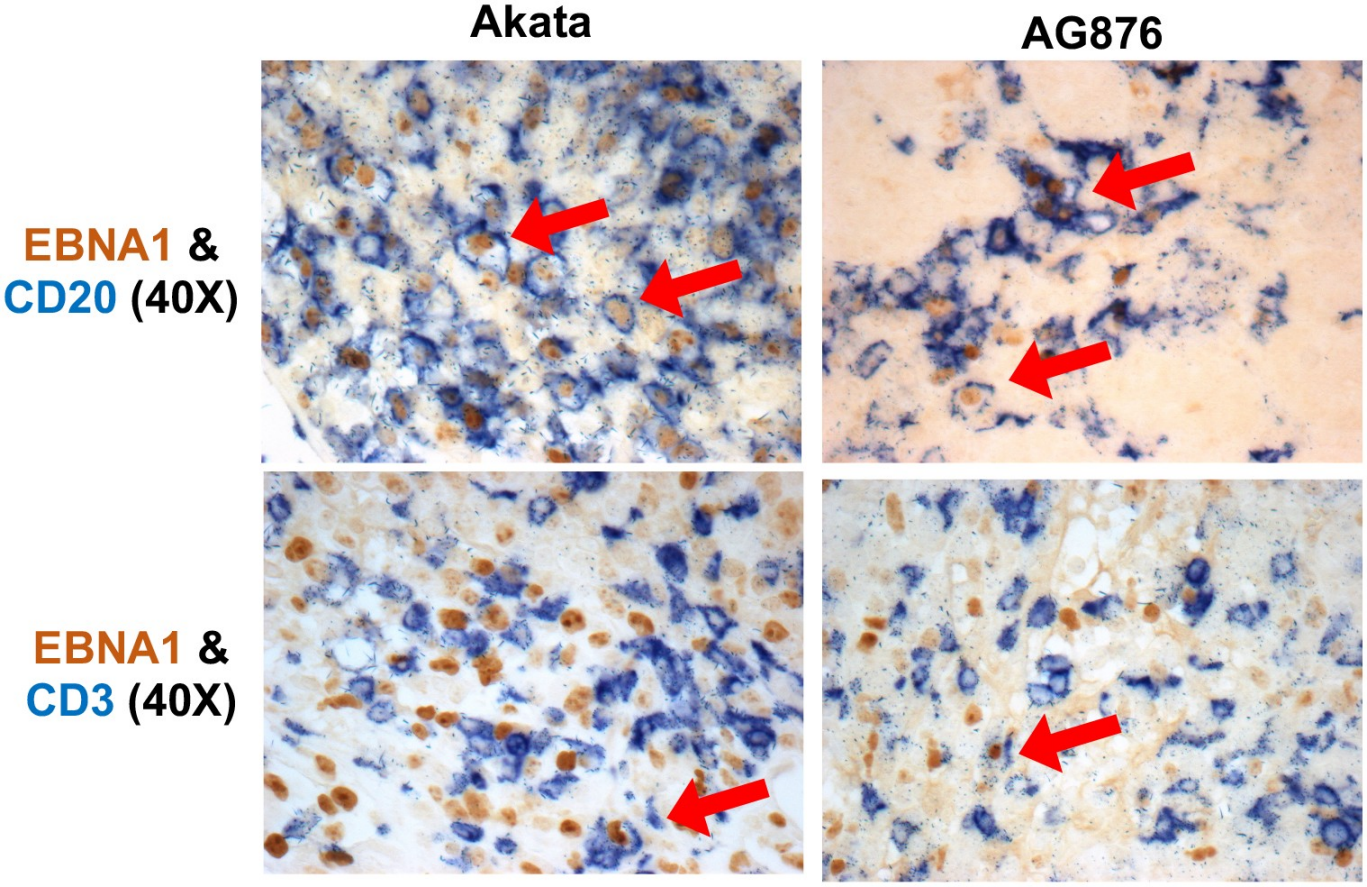
T2 EBV does not show elevated T cell infection in CBH mouse model. IHC co-staining analysis was performed on T1 and T2 EBV-induced lymphomas using antibodies that recognize EBNA1 (EBV latency gene), CD20 (B cell marker) and CD3 (T cell marker) as indicated. Co-staining cells are indicated with the arrows. Only very rare CD3/EBNA1 co-staining cells were identified in lymphomas containing either T1 or T2 EBV.

Since T2 EBV has been reported to infect T cells more efficiently than T1 EBV *in vitro* and *in vivo* [33–35], we also examined whether infection with T2 EBV strains results in more EBV-infected T cells in the CBH mouse model relative to infection with T1 EBV strains. As shown in Fig 4, in IHC co-staining studies of lymphomas, we easily detected EBNA1/CD20 co-staining cells in animals infected with either T1 or T2 viruses, while EBNA1/CD3 co-staining cells were very rare (but present) in lymphomas containing either type of virus. We observed similar results in animals infected with BL5 (T2 EBV) and Mutu (T1 EBV) (S1 Fig). Thus, the great preponderance of EBNA1-expressing cells were B cells (CD20-positive) and not T cells (CD3-positive) in both T1 and T2 EBV-induced lymphomas.

### T2 EBV infection of primary B cells generates LCLs with a reduced growth rate *in vitro*

**Fig 5:**
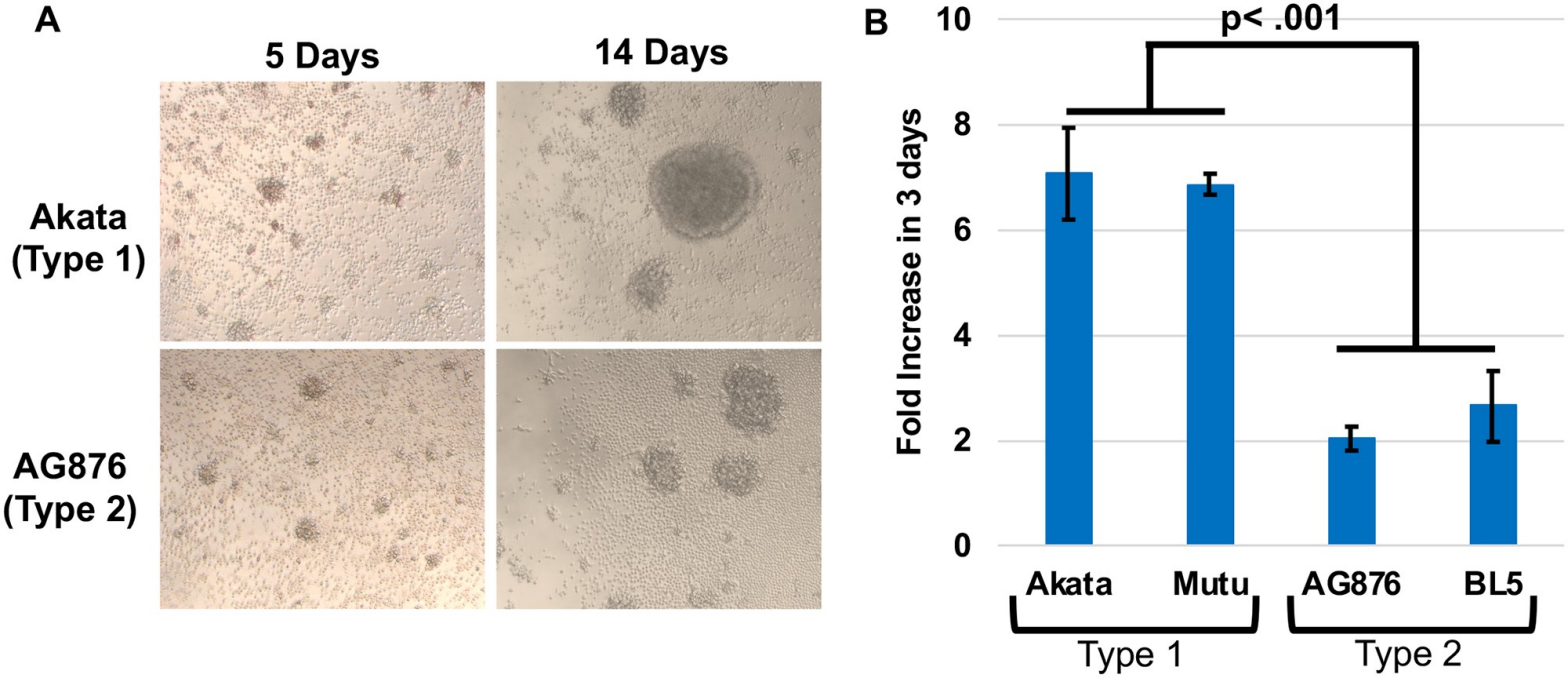
T2 EBV-infected LCLs proliferate at a reduced rate compared to T1 EBV- infected LCLs. **A)** Light microscopy images of T1 and T2 EBV-infected B cells at 5 days and 14 days post infection are shown. **B)** T1 and T2 EBV-infected LCLs were counted 3 days after diluting cells to 1x10^5 cells per mL. The fold increase in cell count was determined by comparing counts at 72 hrs to initial cell number using trypan blue staining. Experiments were performed using three different LCL clones and clones were counted in duplicate. Wilcoxon-Rank Sum Test was performed comparing Type 1 LCLs versus Type 2 LCLs. A p-value < .05 is considered significant.

To determine if the lytic phenotype of T2 EBV is maintained *in vitro*, we generated LCLs by infecting peripheral blood B cells (all from the same donor) using two different T2 EBV strains, AG876 and BL5, and two different T1 EBV strains, Akata (Zp-V3 containing) and Mutu (Zp-P containing). T1 and T2 LCLs were generated successfully using all the virus strains. However, as previously described [30], although B cells infected with T2 EBV strains had similar numbers of blasting cells at early times (day 4) after infection, by 2 weeks post-infection the growth rate of B cells infected with T2 strains was decreased compared to T1 EBV-infected cells (Fig 5A). We examined the fold increase in cell number of T1 EBV-infected versus T2 EBV-infected LCLs over 72 hours using trypan blue counting analysis at 8 weeks post-infection and confirmed that the T2 EBV LCLs have a significantly reduced (2 to 3-fold) growth rate compared to T1 LCLs (Fig 5B). These results are consistent with previous observations comparing the growth of T1 and T2 EBV LCLs [30].

### LCLs infected with T2 EBV have higher levels of lytic viral protein expression in comparison to LCLs infected with T1 EBV

**Fig 6:**
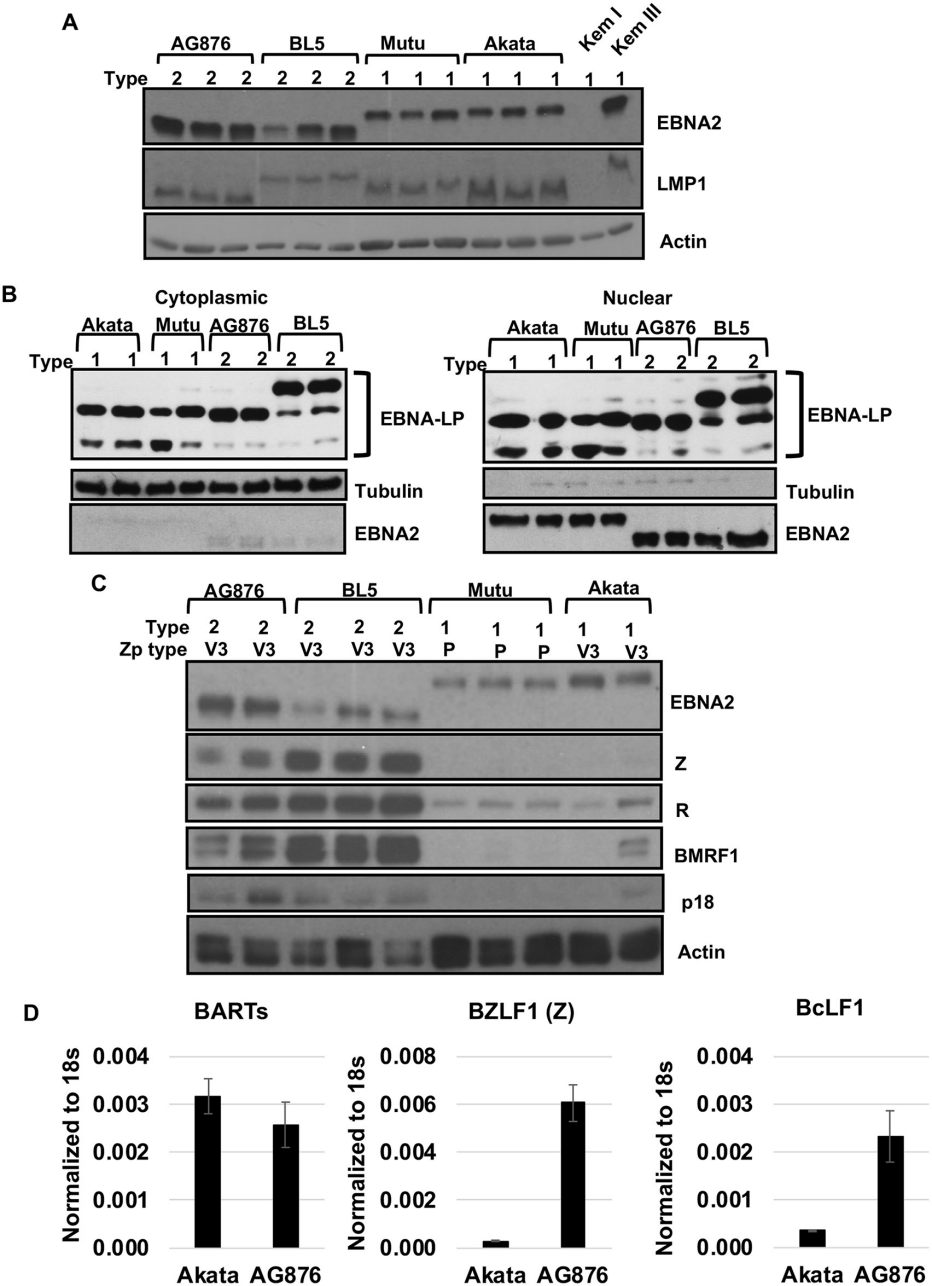
T2 EBV-infected LCLs have increased lytic infection compared to T1 EBV-infected LCLs. **A)** Immunoblot analysis of LCLs (from the same donor) derived from two different T1 strains (Akata and Mutu) and two different T2 strains (AG876 and BL5) was performed using antibodies against EBNA2 (EBV latency protein), LMP1 (EBV latency protein), and actin. Kem I and Kem III serve as negative and positive controls, respectively, for presence of EBNA2 and LMP1. **B)** Nuclear and cytoplasmic extracts from T1 and T2 EBV-infected LCLs were subjected to immunoblot analysis using antibodies against EBNA-LP, Tubulin (cytoplasmic fraction control), and EBNA2 (as a nuclear fraction control). **C)** Immunoblot analysis of LCLs using antibodies against EBNA2, Z (immediate-early lytic protein), BRLF1(R) (immediate-early lytic protein), BMRF1 (early lytic protein), p18 VCA (late lytic protein), and actin. **D)** RNA isolated from T1 (Akata) or T2 (AG876) EBV-infected LCLs was subjected to qPCR analysis using primers against 18S rRNA, BARTs (EBV miRNA transcript), BZLF1 (immediate-early lytic gene), and BcLF1 (late lytic gene). The primers for EBV genes recognize both T1 and T2 EBV; results were normalized to the level of the cellular 18S transcript.

We next performed immunoblot analysis to compare the levels of latent and lytic viral proteins expressed in LCLs infected with T1 EBV versus T2 EBV. No consistent difference in the expression level of the latent EBNA2 protein was observed, although as expected T2 EBV EBNA2 has a lower molecular weight than T1 EBV EBNA2 (Fig 6A). At the time points studied (6–8 weeks after EBV infection), LMP1 expression levels were not consistently different in LCLs infected with T1 versus T2 EBV. Normalization of LMP1 expression between T1 versus T2 LCLs at later time points has been previously described [39] and is attributed to the strong selective pressure for increased LMP1 expression to promote growth of LCLs. Since EBNA-LP is known to enhance EBNA2 activity, including activating LMP1 expression [40–45], we also compared the amount of nuclear and cytoplasmic EBNA-LP in our T1 and T2-infected cells. We observed no consistent difference in EBNA-LP expression in T1 and T2-infected LCLs (Fig 6B), suggesting that differences in EBNA2 function in T1 versus T2 LCLs are not likely to be mediated by differences in the amount, or cellular location, of EBNA-LP.

Importantly, as shown **in** Fig 6C, LCLs infected with either T2 EBV strain expressed considerably higher levels of multiple different early lytic and late lytic viral proteins (including Z, R, BMRF1 and p18VCA) relative to the corresponding LCLs infected with either of two different T1 EBV strains. Quantification of the immediate-early gene BZLF1 transcript and late lytic gene BcLF1 transcript by qPCR in two different AG876-infected LCLs and two different Akata virus-infected LCLs showed higher levels of the BZLF1 and BcLF1 transcripts in AG876 virus (T2 EBV) infected LCLs, although expression of the viral microRNAs (BARTs) was similar (Fig 6D). This result suggests that increased activity of the EBV BZLF1 IE gene promoter (the master regulator of lytic EBV reactivation in B cells) leads to enhanced lytic viral reactivation in T2 LCLs.

### BZLF1 expression does not contribute to the slow growth phenotype of T2 LCLs but instead promotes growth of cells

**Fig 7:**
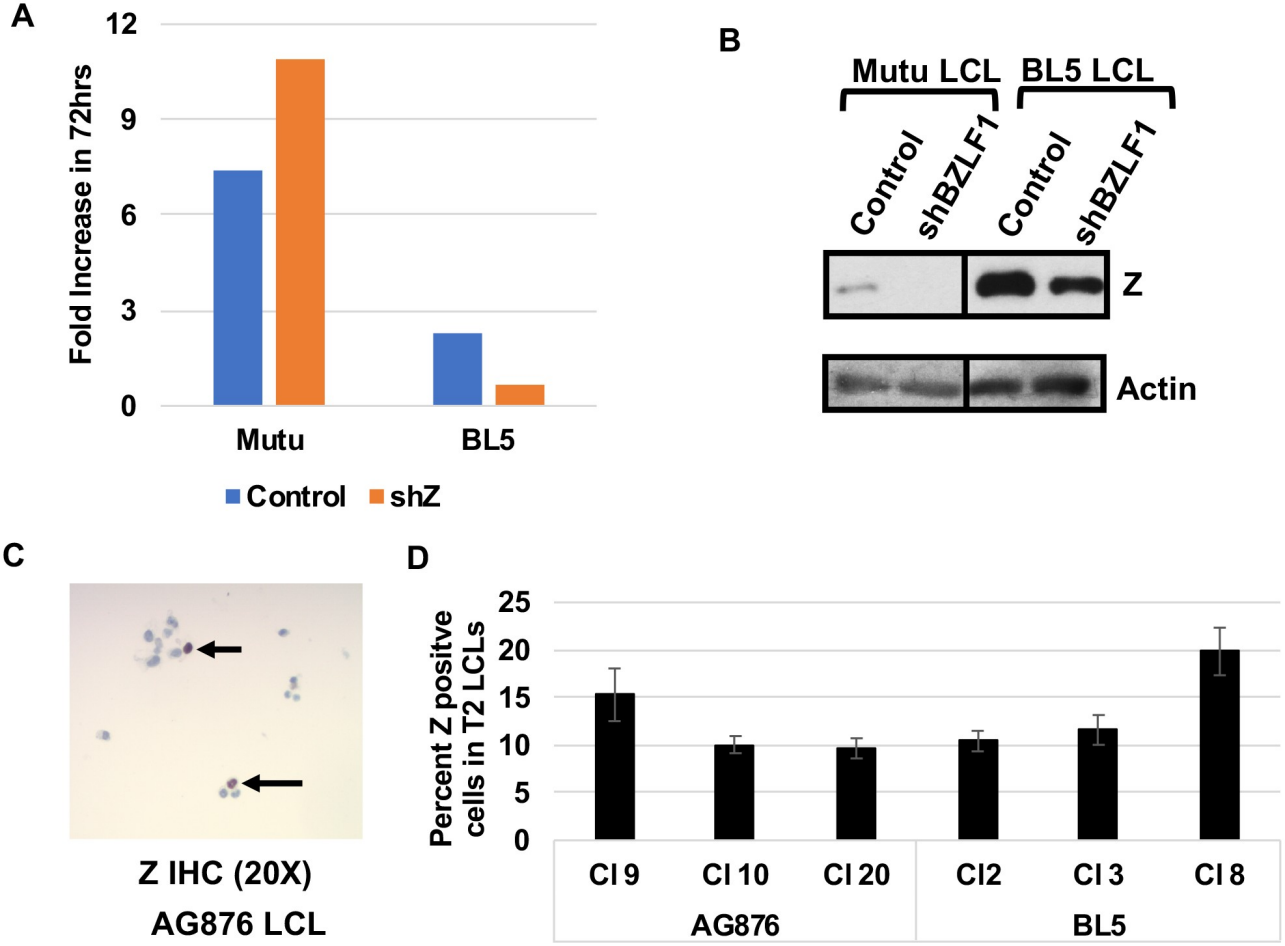
Lytic infection in T2 EBV-infected LCLs does not reduce growth rate. **A)** T1 EBV-infected (Mutu) or T2 EBV-infected (BL5) LCLs stably infected with control shRNA or shRNAs targeting Z were diluted to 1x10^5 cells and counted 72 hours later. The fold increase in cell number was determined by comparing cell counts at 72 hours to initial cell number. Experiment was performed in triplicate. **B)** Immunoblot analysis of BL5 LCLs or Mutu LCLs infected with either control shRNA or shRNA targeting Z using antibodies against Z and actin. **C)** IHC analysis of T2 LCLs was performed using antibody against Z. Arrows indicated Z staining cells. **D)** Quantification of the percent of EBV-infected cells in type 2 LCLs expressing Z as determined by IHC analysis. At least 10 fields of view were quantified across 3 independently derived Type 2 LCL clones infected with either the AG876 or BL5 viruses.

Since lytic EBV infection can kill the host cell through a variety of mechanisms, we considered the possibility that T2 LCLs grow more slowly than T1 LCLs because they are more lytic. To examine this, T2 LCLs were stably infected with a lentivirus expressing an shRNA targeting the BZLF1 gene or a control vector. Unexpectedly, reducing BZLF1 expression in T2 BL5-infected LCLs resulted in a reduction of proliferation in BL5 LCLs (Fig 7A and 7B). Likewise, treatment of AG876 LCLs with a BZLF1 shRNA (which reduced BZLF1 expression to a level similar to that in Mutu LCLs) did not increase cellular proliferation and instead modestly reduced proliferation (S2 Fig). These results suggest that BZLF1 expression may actually promote the survival of T2 EBV-infected LCLs, possibly through the previously described ability of BZLF1 to induce expression of the B-cell growth factor, IL-6 [10]. To compare the number of cells containing latent versus lytic infection in T2 EBV infected LCLs, we used IHC to determine the percentage of AG876 LCLs expressing the IE Z protein. This analysis revealed that the majority of the infected cells (80 to 90%) do not express Z in either AG876 or BL5 LCLs and are latently infected (Fig 7C and 7D); similar results were observed in the T2 EBV-infected lymphomas in CBH mice (Fig 3A). Thus, although B cells infected with T2 EBV are more likely to enter lytic infection in comparison to B cells infected with T1 EBV, the majority of T2 EBV-infected B cells are latently infected in both the CBH model and *in vitro* LCL lines.

### Lytic infection in T1 Akata LCLs can be restored by ionomycin, and constitutive lytic infection in T2 LCLs requires NFAT activity

**Fig 8:**
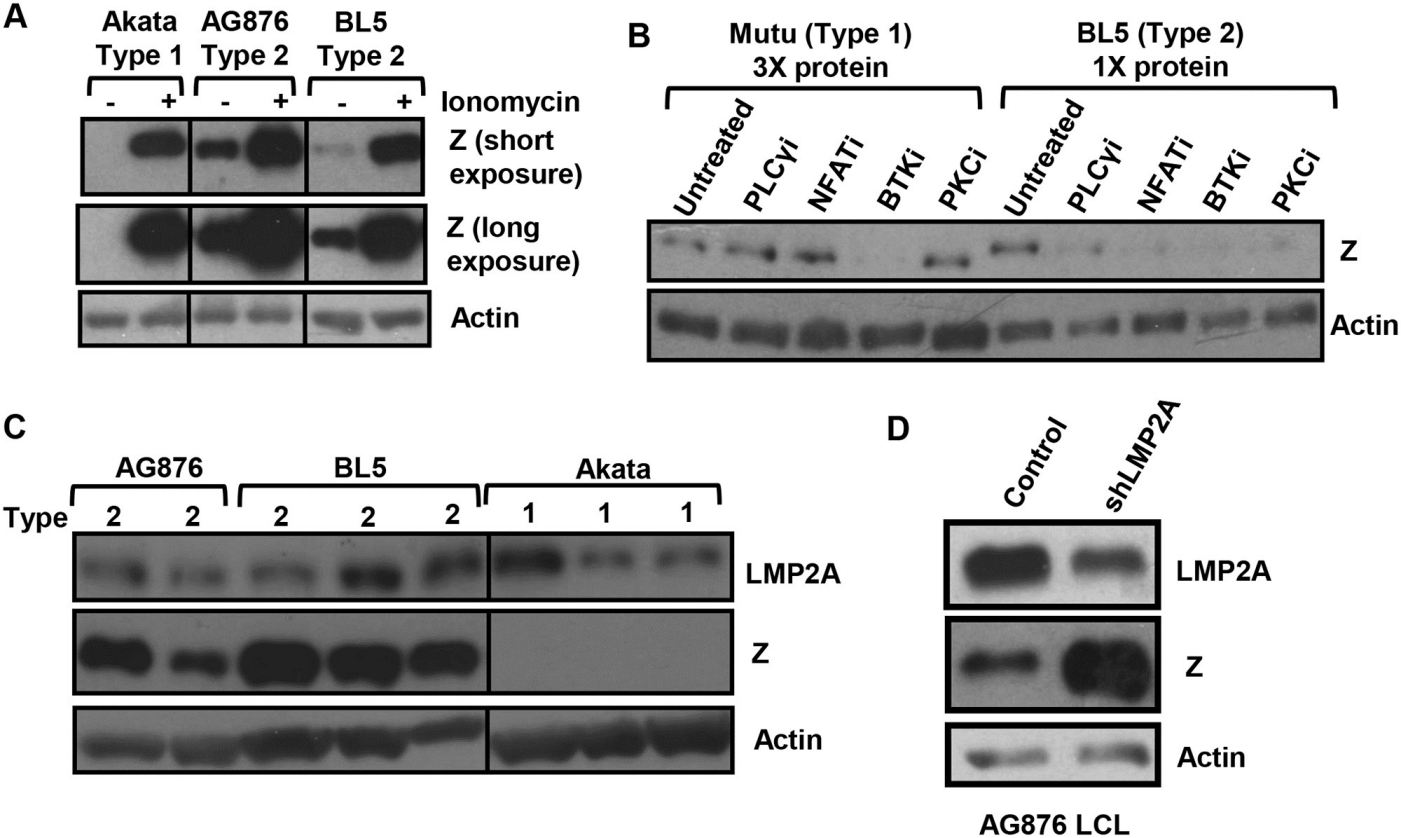
T2 EBV lytic infection depends upon NFAT, PLCγ, BTK, and PKC activity. **A)** T1 and T2 EBV-infected LCLs were treated with ionomycin for 48 hours and immunoblot analysis performed using antibodies that detect EBV Z protein or cellular actin as indicated. **B)** T1 and T2 LCLs were treated with inhibitors that target various components of the BCR pathway, including the PLCγ inhibitor, U73133, the NFAT inhibitor, cyclosporin A, the BTK inhibitor, Ibrutinib, and the PKC inhibitor, PKC412. Extracts were harvested 48 hours and immunoblot analysis was performed using antibodies against the EBV Z protein and actin as indicated. Note that three times more protein is loaded in the T1 Mutu lanes to normalize for differences in the level of constitutive Z expression. **C)**. Immunoblot analysis was performed on whole cell lysates from T1 and T2 EBV-infected LCLs using antibodies against LMP2A (an EBV latency protein), Z, and actin as indicated. **D)** AG876 T2 EBV-infected LCLs were stably transduced with lentiviruses expressing control or LMP2A targeted shRNAs and cell lines were generated. Immunoblot analysis was performed using antibodies against LMP2A (latency gene), Z, and actin as indicated.

We previously showed that the Zp-V3 form of Zp is much more efficiently bound by (and activated by) NFATc1 in comparison to the Zp-P form of the promoter [20]. Since T1 Akata virus has the Zp-V3 form of Zp, yet Akata virus-infected B cells are more latent than T2 EBV-infected B cells (Figs 3A, 6C and 6D), we hypothesized that the amount of one or more activated NFAT family members might be limiting in T1 EBV infected B cells compared to T2 EBV infected B cells. NFAT transcription factors must be dephosphorylated by the calcium-activated phosphatase, calcineurin, in order to translocate to the nucleus and activate target promoters [46,47]. BCR signaling (as well as a number of other signaling pathways) increases intracellular calcium (and hence calcineurin activity and NFAT nuclear translocation) by activating PLCγ2 [48,49]. To determine if increasing calcium flux is sufficient to restore lytic gene expression in Akata LCLs, we treated cells with or without ionomycin, an agent that increases intracellular calcium [50]. Although T1 Akata LCLs have much less constitutive Z expression than T2 AG876 and BL5 LCLs, ionomycin treatment induced Z expression in Akata LCLs to a level similar to that in BL5 cells (Fig 8A). This result suggests that one or more calcium-activated transcription factors may be limiting in T1 Akata LCLs.

We next asked if inhibition of NFAT activity (using the calcineurin inhibitor, cyclosporine) decreases the amount constitutive lytic infection in T2 BL5 cells. As shown in Fig 8B, cyclosporine treatment blocked constitutive lytic infection in BL5 LCLs. Similar results were observed in AG876 LCLs (S3A Fig). In contrast, cyclosporine did not decrease the amount of BZLF1 expression in a Mutu LCL clone that (unlike most clones) had some degree of constitutive lytic Z expression; note that three times as much protein is loaded for Mutu cells versus BL5 cells on the western blot shown to normalize for levels of Z expression levels in the untreated cells. These results indicate that T2 LCLs (but not Mutu T1 LCLs) require NFAT activity for their constitutive Z expression.

Since BCR signaling is thought to induce NFAT activity through a PLCγ2 pathway, and the ability of BCR signaling to reactivate EBV in Akata virus-infected Burkitt lymphoma cells requires both BTK and PKC activity [48,49,51,52], we also compared the effect of PLCγ, BTK and PKC inhibitors on the amount of constitutive lytic Z expression in BL5 cells versus Mutu cells. As shown in Fig 8B, all three types of inhibitors reduced constitutive Z expression in the T2 LCL, consistent with cellular BCR signaling playing a role in activation of the Zp-V3 Z promoter in T2 BL5 LCLs. We also observed similar results in AG876 LCLs (S3A Fig). In contrast, only the BTK inhibitor reduced constitutive Z expression in Mutu LCLs (most likely through alternative mechanism(s)).

In addition, since the EBV latent LMP2A protein can mimic the effect of low level tonic BCR signaling, we compared the level of LMP2A expression in T1 Akata LCLs versus T2 BL5 and AG876 LCLs (Fig 8C). We found no consistent difference in LMP2A expression; although LMP2A protein encoded by Mutu virus could not be recognized by the antibody used. We also observed no consistent differences between Type 1 LCLs containing B95.8 and Akata when compared to AG876 and BL5 (S3B Fig). Interestingly, when LMP2A expression was decreased using an shRNA in a T2 AG876-infected LCL, we observed an increase in the amount Z expression (Fig 8D). Thus, LMP2A acts as an inhibitor of lytic reactivation in the Type III latency environment of LCLs, consistent with previously published data showing that LMP2A inhibits the authentic BCR stimulation [53–55]. Therefore the NFAT-dependent constitutive lytic infection in T2 LCLs is more likely derived from signals emanating from the authentic BCR rather than LMP2A.

### T2 EBV-infected LCLs have increased NFATc1 and NFATc2 compared to T1 EBV-infected LCLs

**Fig 9:**
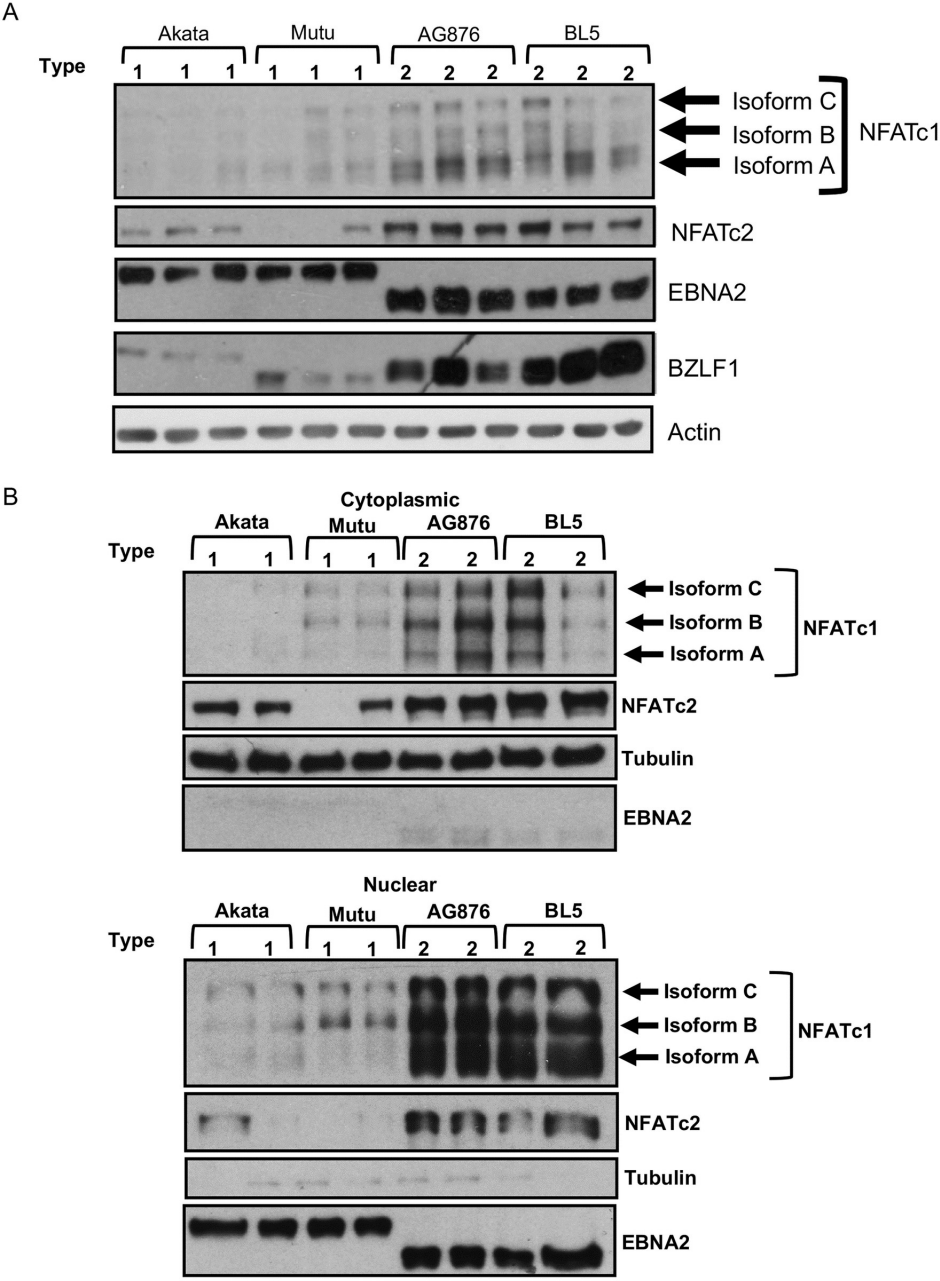
T2 EBV-infected LCLs have elevated total and activated (nuclear) NFATc1 and NFATc2 compared to T1 EBV-infected LCLs. **A)** Whole cell extracts from T1 and T2 EBV-infected LCLs were subjected to immunoblot analysis using antibodies against NFATc1, NFATc2, EBNA2, Z, and actin. **B)** Cellular fractionation was performed on T1 and T2 LCLs and fractionation samples were subjected to immunoblot analysis. **Upper)** Immunoblot analysis performed on cytoplasmic fraction using antibodies against NFATc1, NFATc2, EBNA2 (nuclear fraction control), and Tubulin (cytoplasmic fraction control). **Lower)** Immunoblot analysis performed on nuclear fraction using antibodies against NFATc1, NFATc2, EBNA2 (nuclear fraction control), and Tubulin (cytoplasmic fraction control).

Given that calcineurin activity is required for constitutive Z expression in T2 AG876 cells but not T1 Mutu cells, we asked whether LCLs with T2 EBV have increased amounts of nuclear NFATc1 and/or NFATc2 in comparison to T1 LCLs. Although we previously showed that NFATc1 plays a role in mediating ionomycin-induced lytic EBV reactivation in Mutu Burkitt lymphoma cells infected with a Zp-V3 containing mutant B95.8 virus [20], we have not previously explored whether the related NFATc2 protein is expressed in LCLs or Burkitt lines, and whether this factor is also required for Zp-V3 activation by calcium signaling pathways. As shown in Fig 9, T2 LCLs (AG876 and BL5) have a much higher level of both total and nuclear NFATc1 (especially the shortest isoform, whose promoter is driven by BCR activation [46]) compared to T1 LCLs. Furthermore, the level of total NFATc2, as well as nuclear NFATc2, is also strikingly increased in T2 LCLs versus T1 LCLs (Fig 9). This result suggests that enhanced expression and activation of both NFATc1 and NFATc2 in T2 LCLs may play a major role in mediating enhanced constitutive Z expression.

### T2-infected LCLs have similar levels of calcium as T1-infected LCLs

**Fig 10:**
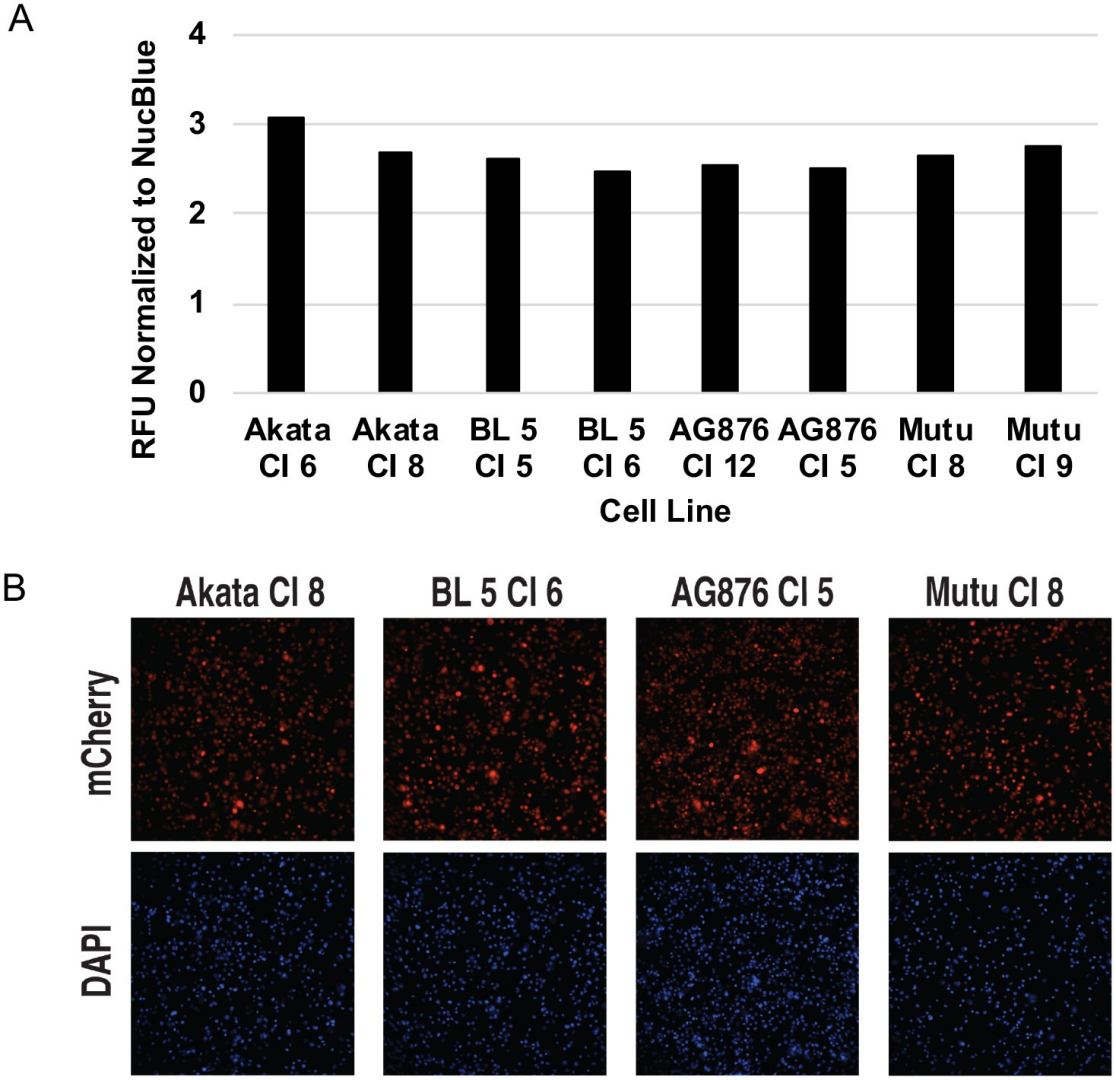
Calcium levels in T2 EBV-infected LCLs are similar to T1 EBV-infected LCLs. Using a fluorescent dye that increases in intensity with elevations in cellular calcium concentration, T1 and T2 LCLs were stained with the dye as described in the methods. **A)** Quantitative imaging analysis was performed using FIJI/ImageJ software and fluorescent intensity was normalized to the intensity of a nuclear control dye (NucBlue) as indicated. **B)** Representative fluorescent images for each of the different LCLs generated are shown for Calcium and NucBlue.

Since both NFATc1 and NFATc2 are activated by dephosphorylation in a calcium-dependent mechanism, we hypothesized that there may be elevated calcium levels in the T2 infected LCLs. Using a fluorescent dye that increases intensity in response to increased calcium concentration, we compared the levels of constitutive calcium concentration in T1 and T2-infected LCLs. We observed no consistent difference in the amount of constitutive calcium concentration between T1 and T2-infected LCLs (Fig 10). Therefore, the increases in the total amounts of NFATc1 and NFATc2 may explain the enhanced nuclear accumulation of NFATc1/NFATc2 rather than increases in calcium levels per se.

### NFATc2 and NFATc1 cooperate with CAMKIV to induce lytic EBV infection in BL cells infected with Zp-V3 containing EBV

NFATc1 and NFATc2 share the ability to bind to the NFAT binding motif, but do not always affect promoters containing NFAT binding sites in the same manner [56]. Furthermore, while NFATc1 inhibits the ability of BCR activation to induce apoptosis in Burkitt lymphoma cells, NFATc2 has the opposite effect [56–58]. Predicting the effects of NFATc1 on a promoter are further complicated by the fact that the short form of NFATc1 is driven by a different (BCR-responsive) promoter than the other two isoforms, and that different isoforms of NFATc1 sometimes have different effects on the same promoter [46,56,59].

Since we previously showed that a constitutively active form of the short isoform of NFATc1 can activate the Zp-V3 form of the Z promoter in reporter gene assays [20], we examined whether a vector expressing a constitutively active form of NFATc2 can induce lytic EBV reactivation in Akata virus-infected Burkitt lymphoma cells in the presence or absence co-transfected constitutively active (short isoform) NFATc1 and/or constitutively active CAMKIV vectors. The combination of constitutively active CAMKIV (which phosphorylates and activates c-Jun and MEF2D, both of which can bind to Zp), and calcineurin (which activates NFATc1/NFATc2) was previously shown to be sufficient to induce lytic reactivation in Akata Burkitt cells, although neither vector alone was effective [60–62]. Interestingly, LMP1 activates CAMKIV expression in B cells, and LCLs (but not Burkitt cells) express CAMKIV [60].

**Fig 11:**
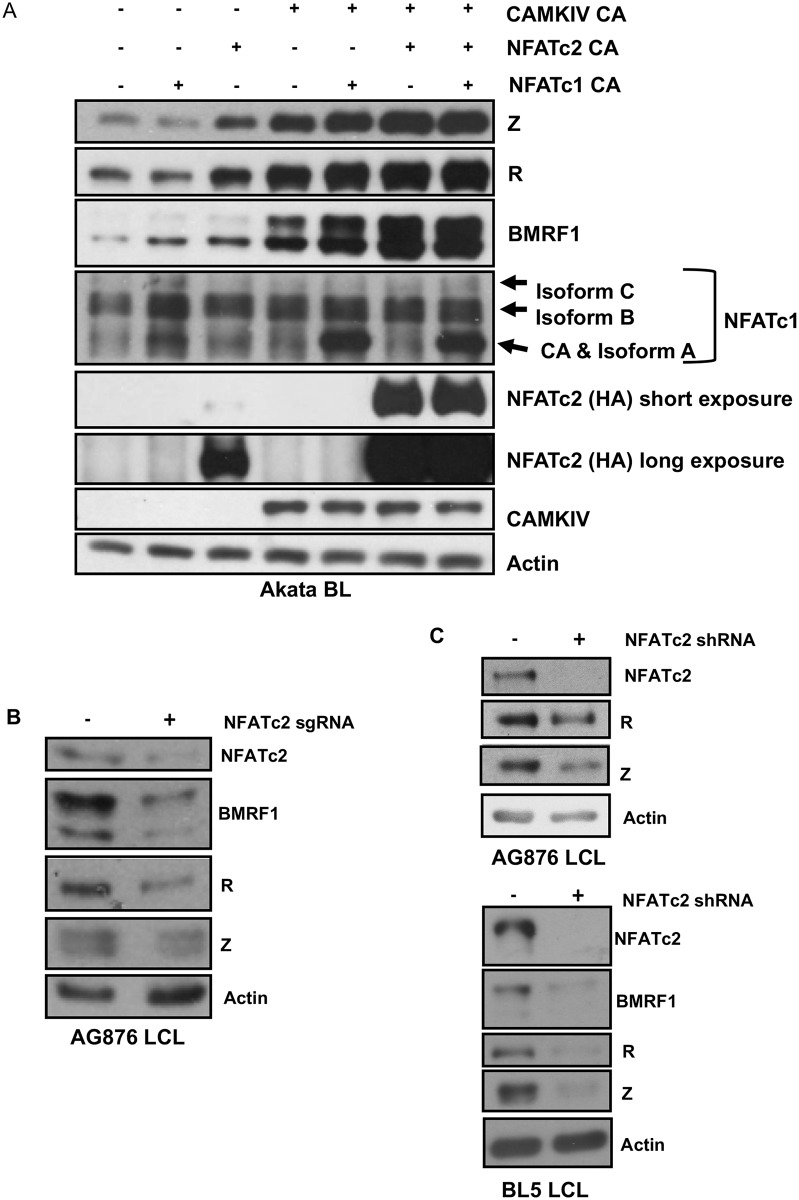
NFATc1 and NFATc2 collaboratively increase lytic infection in the presence of CAMKIV in Akata Burkitt lymphoma cells. **A)** Akata BL cells were nucleofected with plasmids containing constitutively active NFATc1, NFATc2, and CAMKIV either alone or in combination as indicated. Cell extracts were subjected to immunoblot analysis 48 hours later using antibodies against BZLF1, BMRF1, NFATc1, HA (which recognizes tagged NFATc2), CAMKIV, and actin. **B)** AG876 LCLs were transfected with an NFATc2 directed sgRNA/Cas9 complex as indicated. Transfected cells were subjected to immunoblot analysis using antibodies against NFATc2, BMRF1, Z, R, and actin as indicated. **C)** AG876 and BL5 type 2 LCLs were stably infected with a lentivirus containing a shRNA against NFATc2, selected in puromycin for 5 days, then subjected to immunoblot analysis using antibodies against NFATc2, R, Z, BMRF1 and actin.

As shown in Fig 11A, constitutively active NFATc2 induced expression of the Z, R, and BMRF1 lytic proteins in Akata Burkitt cells, particularly when combined with a constitutively active CAMKIV vector. The constitutively active NFATc1 (short isoform) only induced lytic gene expression when in combination with the constitutively active CAMKIV, although not to the same degree as NFATc2 when combined with CAMKIV. The most effective combinations for inducing Z and BMRF1 expression in Akata Burkitt cells was the combination of CAMKIV and NFATc2 and the triple combination of CAMKIV, NFATc2, and NFATc1. Thus, NFATc1 and NFATc2 likely collaborate with CAMKIV to induce lytic EBV reactivation through a calcineurin-dependent manner in B cells infected with ZpV3-containing EBV strains.

To further address whether NFATc2 is a positive regulator of lytic infection in the context of T2 EBV-transformed LCLs, we knocked down NFATc2 in lytic type 2 LCLs using either a CRISPR/Cas9 approach or an NFATc2 specific shRNA (Fig 11B and 11C). Using either approach, decreased NFATc2 expression was associated with a decrease in the amount of lytic EBV protein expression (Fig 11B and 11C). We observed at least a two-fold decrease in Z, R, and early lytic protein BMRF1 expression when we knocked-down NFATc2 expression in type 2 EBV-infected LCLs (S4 Fig). Together, these results indicate that increased levels of both activated NFATc1 and activated NFATc2 contribute to the enhanced level of lytic EBV infection in T2 LCLs.

### Differences in the T1 versus T2 EBNA2 proteins are not sufficient to alter the level of lytic EBV protein expression in the context of Burkitt lymphoma B cells

**Fig 12:**
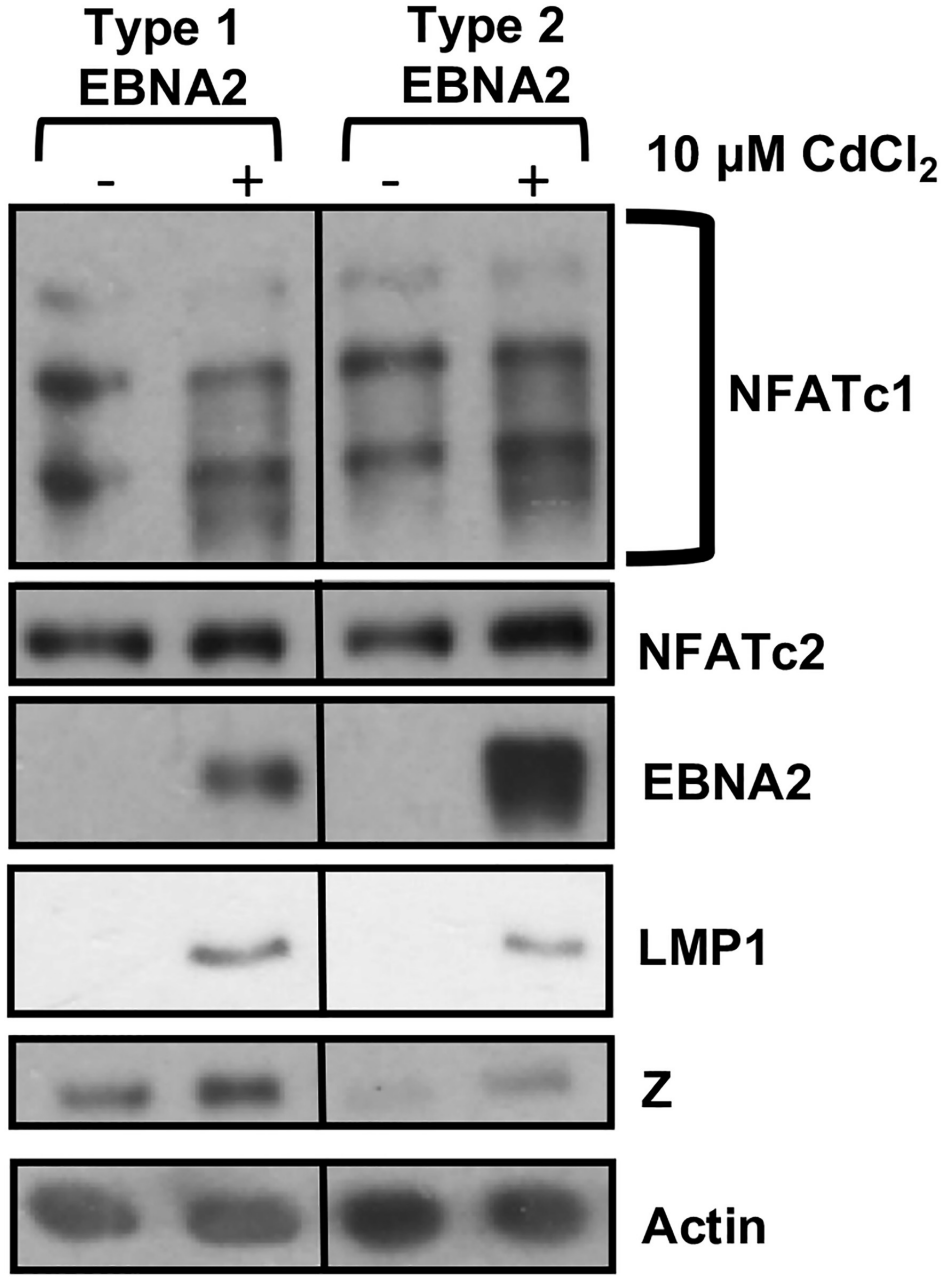
T1 and T2 EBNA2 expression have similar effects on NFATc1/NFATc2 expression in P3HR1 Burkitt lymphoma cells. P3HR1 cells containing either T1 or T2 pHEBo-MT-EBNA2 constructs were treated with or without 10 μM CdCl_2_ for 48 hours to induce EBNA2 expression. Immunoblot analysis was performed on whole cell extracts using antibodies against NFATc1, NFATc2, EBNA2, LMP1, Z, and actin.

To determine if any of the differences observed in T1 versus T2 EBV infected LCLs can be attributed to differences in the T1 versus T2 EBNA2 functions, we expressed cadmium-inducible T1 EBNA2 or T2 EBNA2 proteins in the P3HR1 Burkitt lymphoma line [28]. The P3HR1 virus is a “Wp-restricted” T2 EBV strain that is deleted for the EBNA2 gene but still expresses EBNA3 proteins [63]. When EBNA2 was induced using cadmium chloride, we observed reduced expression of LMP1 in cells expressing T2 EBNA2 relative to cells expressing T1 EBNA2, as previously described [28]. However, we did not find that expression of either T1 or T2 EBNA2 affects NFATc1 or NFATc2 expression in the Burkitt P3HR1 cell environment (in which NFATc1 and NFATc2 are already relatively highly expressed), and cells with T2 EBNA2 did not have increased Z expression relative to cells with T1 EBNA2 (Fig 12). Therefore, differences in EBNA2 function may not be solely responsible for differences in the level of NFATc1/NFATc2/Z expression that occur in LCLs with T1 versus T2 EBV infection. Nevertheless, it is also possible that effects of the T1 versus T2 EBNA2 proteins on NFATc1/NFATc2 expression are specific to the LCL environment and cannot be observed in the Burkitt cell environment.

## Discussion

Although it has long been known that two major types of EBV exist, relatively little is known regarding potential differences in the behaviors of these different viruses in humans. The main phenotypic difference *in vitro*, T1 EBV’s ability to more efficiently transform B cells into long-term LCLs, maps to a single amino acid alteration in EBNA2, and is correlated with a reduction in the ability of T1 EBNA2 to increase LMP1 expression [28]. However, the biological and clinical relevance of this phenotypic difference *in vivo* remains unclear, since the frequency of T2 versus T1 EBV-derived human lymphomas appears to be similar to the frequency of T2 versus T1 EBV infection in healthy individuals [21,25,64]. Our studies here used both a cord blood-humanized *in vivo* mouse model, and *in vitro* derived LCLs, to compare the phenotypes of T1 versus T2 EBV strains. Our results show that a major previously unappreciated phenotype of T2 EBV strains is their enhanced ability to undergo the lytic form of viral infection in B cells. Since we previously showed that Zp-V3 contains an NFAT binding motif and responds more efficiently than prototype Zp-P to BCR signaling, one reason for the increased lytic infection in T2 LCLs is the universal presence of the Zp-V3 form of the BZLF1 IE promoter in T2 strains [20,23,24]. In addition, we have now discovered that increased activated NFATc1 and NFATc2 in T2 LCLs is also important for the lytic phenotype. Together our results suggest that T2 EBV has evolved to use multiple different strategies to ensure a more lytic life cycle in B cells whereas T1 EBV has evolved to ensure efficient viral latency in B cells.

Consistent with previous *in vitro* studies, we found that T2 EBV-infected lymphomas in our cord blood-humanized mouse model express less LMP1 in comparison to T1 EBV-infected lymphomas. Nevertheless, we did not find that T2 EBV strains (AG876 and BL5) are obviously impaired for the ability to form lymphomas in this *in vivo* model. This result is not particularly surprising, since we recently showed that an LMP1-deleted T1 virus (B95.8) can form lymphomas in the CBH model and demonstrated that CD40 ligand-expressing CD4 T cells can at least partially replace the requirement for LMP1 expression by providing an alternative source of CD40 ligand signaling [5]. Another group also recently found that the T2 LCL-10 EBV strain induces lymphomas in a humanized mouse model with a similar efficiency as a T1 EBV strain (B95.8) [33]. In this paper, we have extended the previous work by examining the phenotypes of a variety of different T1 versus T2 EBV strains and comparing the effects of each virus type using different infectious virus titers. We conclude that in spite of the reduced transformation efficiency of T2 EBV strains *in vitro*, both T1 EBV and T2 EBV strains are tumorigenic in the CBH model, and result in activated DLBCLs with similar histologic phenotypes. Furthermore, both T1 and T2 tumors arise in similar organs, in particular the pancreas, liver, and biliary track. In addition, both T1 and T2 tumors are heavilty infiltrated with T cells.

Although T2 EBV is reported to have an increased ability to infect T cells both *in vitro* and *in vivo* [33–35], we were unable to show evidence of a clear increase in T cell infection by T2 EBV in this model. Similar to the IHC co-staining results, our analysis of purified CD3+ cells from peripheral blood of T1 versus T2 EBV infected CBH mice (using qPCR methods to detect various EBV transcripts) did not reveal significant EBV infection of T cells by either virus type. The absence of significant T cell infection by T2 EBV in the CBH mouse model (at least at later time points after infection) may indicate that T cell infection is very transient (perhaps quickly killing the cells) and/or that EBV preferentially infects a T cell type (for example immature T cells) that are not well represented in this model. In any event, the lytic infection observed in animals infected with T2 EBV in this model largely occurs in B cells rather than T cells.

One reason for the highly lytic infection found in T2 EBV-infected B cells is the universal presence of the Zp-V3 form of the BZLF1 promoter (which is more responsive to NFAT-inducing stimuli) in all T2 EBV strains [20,23,24]. Nevertheless, the presence of the Zp-V3 promoter variant by itself appears to not be sufficient to ensure high level lytic infection in all contexts, since we found that in EBV-infected B cells, the two T2 EBV strains (AG876 and BL5) were still more lytic compared to two T1 EBV strains (Akata and M81) that also contain the Zp-V3 variant (Figs 3B and 6C). Interestingly, another potential mechanism for increased lytic infection could have been due to differences in EBER2, since it has been previously reported that the EBER2 gene potentiates lytic infection in the case of the M81 strain of EBV [65]. In our study, since AG876 and Akata EBV strains have identical EBER2 sequences, this is not a likely mechanism. We thus hypothesized that additional trans-acting viral and/or cellular factors that differ between T1 versus T2 EBV-infected B cells must also contribute to the lytic phenotype in B cells with T2 infection.

We initially considered the possibility that differences in the expression and/or sequences of T1 versus T2 LMP2A and/or LMP1 might contribute to the lytic phenotype of T2 LCLs, since LMP2A mimics the effect of low level tonic BCR signaling and LMP1 has been shown to suppress lytic EBV reactivation in B cells [37,53–55]. However, AG876 (T2 EBV) and Akata (T1 EBV) have the same LMP2A sequence, and we did not find consistent differences in the level of LMP2A expression in T1 versus T2 B cells (Fig 8C). Furthermore, at least in the context of Type III latency, LMP2A may be an inhibitor of lytic infection in T2 LCLs, since we found that knockdown of LMP2A increased Z expression (Fig 8D). In the case of LMP1, although we consistently observed decreased LMP1 expression in T2 EBV-infected lymphomas in CBH mice *in vivo*, we found that over time T2 EBV LCLs *in vitro* expressed similar levels of LMP1 as the T1 EBV lines (Fig 6), yet still retained the enhanced lytic phenotype. Thus, differences in LMP1 expression do not explain why T2 EBV LCLs remain more lytic than T1 EBV LCLs (at least for the first few months in culture) but do suggest that there is considerably more selective pressure for LCLs with T2 EBV infection to express higher levels of LMP1 compared to lymphomas with T2 EBV infection in the CBH model.

Instead, our results point to differences in the expression and activation of NFATc1 and NFATc2 as a primary mechanism driving enhanced lytic infection in T2 EBV-infected B cells. NFAT transcription factors are activated via calcineurin-mediated dephosphorylation (allowing nuclear translocation) in response to a variety of different calcium inducing signals, and activation of PLCγ2 is required for the ability of BCR signaling to activate calcineurin [46,47,56]. Since we found that both a calcineurin inhibitor and a PLCγ2 inhibitor decreased constitutive expression of the Z IE protein in T2 LCLs, while having little effect on Z expression in a rare T1 LCL clone where Z expression was observed (Fig 8B), constitutive expression of Z in T2 LCLs appears to involve a PLCγ2-dependent, calcineurin-dependent and NFAT-dependent pathway. Most importantly, we discovered that T2 LCLs express higher levels of both total and nuclear NFATc1 and NFATc2 in comparison to T1 LCLs (Fig 9). We then demonstrated that NFATc1 and NFATc2 (in conjunction with CAMKIV) collaboratively activate lytic EBV protein expression in T1 Zp-V3-containing Akata Burkitt cells (Fig 11A). Furthermore, we showed that short-term knockdown of NFATc2 expression in T2 LCLs results in decreased lytic protein expression (Fig 11B and 11C). Thus, we propose that increased activated NFATc1 and NFATc2 expression both contribute to the enhanced lytic phenotype of T2 LCLs. Interestingly, we have been unable to successfully knock-down NFATc1 expression in T2 LCLs, suggesting that NFATc1 serves as growth and/or survival factor for these cells. Likewise, knockdown of Z expression in T2 LCLs decreased the growth rate of T2 LCLs (Fig 7A), suggesting that the lytically-infected cells (approximately 10% of the total T2 LCL cell population (Fig 7B) may promote the growth of the latently infected cells via paracrine mechanisms such as enhanced release of the B-cell growth factor, IL-6 [10].

An important as yet unanswered question is the identity of the viral protein(s) and/or RNAs that mediate the difference in lytic phenotype in T1 versus T2 EBV-infected B cells. Interestingly, we found that differences in T1 versus T2 EBNA2 function are not sufficient to induce changes in NFATc1 or NFATc2 expression in the context of BL cells containing the EBNA2-deleted T2 P3HR1 virus. Nevertheless, we cannot exclude the possibility that EBNA2 type-dependent regulation of NFATc1/NFATc2 expression is specific to LCLs and not present in the context of Burkitt lymphoma cells. Indeed, low levels of IRF4 in Burkitt lymphoma cells may render the ability of T1 EBNA2 to bind to ets-IRF4 combined elements (EICE sites) of little functional consequence [29,31]. Difference in the functions of the T1 versus T2 EBNA3 proteins are another likely mechanism by which the EBV type specific differences in lytic phenotype might occur. In addition, since a recent study (Chen et al) demonstrated the importance of miR-BHRF1-2-5p and miR-BART-2-5p in regulating EBV reactivation [66], it remains possible that differences in the sequences of type 1 versus type 2 miRNAs help to mediate the lytic phenotype. A multiple sequence alignment of 241 EBV genome sequences (Fig S1 of Bridges et al 2019), including 24 type 2 EBV strains (based on their EBNA3 genes), showed that the sequences for these two mature miRNAs are invariant in the alignment [23,67]. This indicates that variation in these mature miRNAs is not likely to mediate the differences in reactivation observed between type 1 and type 2 EBV but we can not preclude potential sequence differences during the immature stage of the BARTs miRNAs.

An intriguing question in the EBV field has been why two different types of EBV have both been successfully maintained in the human population, particularly given the enhanced ability of T1 EBV to transform B cells (at least *in vitro*). Our findings here suggest that any partially impaired ability of T2 EBV to establish long-term viral latency in B cells may be offset by its increased ability to lytically reactivate. Furthermore, in dually-infected B cells, BZLF1 protein produced by a T2 EBV genome could likewise promote reactivation of a T1 EBV genome. Finally, since oropharyngeal epithelial cells are also an important site of lytic EBV replication in humans, it will be important in future studies to compare the phenotypes of T1 versus T2 EBV in this cell type.

## Materials and methods

### Ethics statement

All animal work experiments were approved by the University of Wisconsin-Madison Institutional Animal Care and Use Committee (IACUC) and conducted in accordance with the NIH Guide for the care and use of laboratory animals (protocol numbers M005197 and M005214). We anesthetized mice using isoflurane and euthanized animals by performing cervical dislocations on anesthetized mice [4].

### Cell lines

Akata, originally derived by the Takada lab [68], is a Burkitt lymphoma line containing the T1 Akata strain. The Akata-Bx1 Burkitt cell line (a gift from Lindsey Hutt-Fletcher) was created as previously described [69] and contains a G418 resistance gene and GFP gene inserted into the BXLF1 thymidine kinase gene site in the Akata EBV genome. EBV-negative Akata Burkitt lymphoma cells were a gift from Jeff Sample at Penn State University [70]. Mutu-1, originally derived by the Rickinson lab at the University of Birmingham, UK [71], is a Burkitt lymphoma cell line containing T1 EBV (obtained as a gift from Jeff Sample). AG876 and P3HR1, originally derived by Pizzo *et al* and *Hinuma et al* respectively, are Burkitt lymphoma cell lines containing T2 EBV and were obtained as gifts from Dr. Bill Sugden at the University of Wisconsin Madison [72,73]. BL5, originally derived by the Harrington lab, is a Burkitt lymphoma cell line containing T2 EBV and was obtained as a gift from Rosemary Rochford at the University of Colorado [34,74]. All BL cell lines were maintained in RPMI media containing 10% fetal bovine serum (FBS), and 1% penicillin-streptomycin (pen/strep); Akata-Bx1 cells also were treated with 500 μg per ml of G418. HEK293 cells latently infected with the T1 M81 EBV strain bacmid were derived as previously described [75] and were a gift from Dr. Henri-Jacques Delecluse at Deutsches Krebsforschungszentrum (DKFZ) (via Sankar Swaminathan, University of Utah); these cells were maintained in DMEM containing 10% FBS, 1% pen-strep, and 100 μg/ml of hygromycin B. EBV positive LCL lines were obtained by transforming peripheral blood B from a single donor cells with T1 (Akata and Mutu) and T2 (AG876 and BL5) EBV strains as described below. B95.8 LCLs were a gift from Dr. Bill Sugden (University of Wisconsin-Madison). T1 and T2 EBV LCLs were grown in RPMI media containing 10% fetal bovine serum (FBS) and 1% penicillin-streptomycin (pen/strep).

### Chemical reagents

T1 and T2 LCLs were treated with various drugs which are known to inhibit components of the BCR signaling pathway, including a PLCγ inhibitor, U73133 (Santa Cruz, catalog number: U-73122), a calcineurin inhibitor, Cyclosporin A (Tocris, catalog number: 1101), a BTK inhibitor, Ibrutinib (a gift from Dr. Lixin Rui at the University of Wisconsin), and a PKC inhibitor, PKC412 (Tocris, catalog number: 2992) at doses of .3 μM, 1 μM, 10 μM, and 1 μM respectively for 24–48 hours [20,76–78]. Ionomycin (Sigma, catalog number: I0634), which increases intracellular calcium, was used to induce lytic infection in T1 and T2 LCLs at a dose of 2.5 μg/mL for 24 hours [20].

### shRNA lentiviral vectors and packing lentiviral constructs

Lentivirus vectors expressing short hairpin RNAs (shRNAs) against the immediate-early gene BZLF1 were generated using oligos listed in Table 2. Oligos were annealed by incubating at 95°C for 4 minutes and then allowed to cool to room temperature. Annealed oligos were inserted into the pLKO.1 vector between the AgeI and EcoR1 cut sites [79]. Clones were screened and sequenced confirmed using the pLKO.1 sequencing primer (5’-CAAGGCTGTTAGAGAGATAATTGG-3’). LMP2A shRNA was a gift from Dr. Kathy Shair (University of Pittsburg) [80]. NFATc2 shRNA was purchased from Dharmacon (catalog number: RHS4533-EG4773). Lentivirus was produced as previously described [81]. Briefly, 293T cells were transfected with the lentivirus expression plasmids and the packaging plasmids psPAX2 (Addgene plasmid 12260, a gift from Dr. Didier Trono through Dr. Bill Sugden, University of Wisconsin-Madison) and VSVG (Addgene plasmid 8454, a gift from Dr. Bob Weinberg through Dr. Bill Sugden) using Lipofectamine 2000 reagent (Invitrogen). Supernatant was collected 2 days post-transfection and filtered through a 0.8-μm filter. T1 and T2 EBV LCLs were then transduced by incubation with 8 μg/mL of polybrene for 15 minutes and then centrifuged with lentivirus for 90 minutes at 450g. After 3 days post-infection, stable cell lines were selected with .5 μg/ml puromycin. LCLs were continuously passaged with .5 μg/ml puromycin for up to 4 weeks before lysates were collected, and cell proliferation studies were performed.

**Table 2:** The oligos used to generate BZLF1 shRNA.

Name	Oligo
Forward Oligos	
shRNABZLF1.1F	5' CCGG - AACCCAGAATCAACAGACTAACC CTCGAG GGTTAGTCTGTTGATTCTGGGTT - TTTTTG 3'
shRNABZLF1.2F	5' CCGG - AACCTGGAGACAATTCTACTGTT CTCGAG AACAGTAGAATTGTCTCCAGGTT - TTTTTG 3'
shRNABZLF1.3F	5' CCGG - AATGCTTATCAAGCTTATGCAGC CTCGAG GCTGCATAAGCTTGATAAGCATT - TTTTTG 3'
Reverse oligos	
shRNABZLF1.1R	5’ AATTCAAAAA - AACCCAGAATCAACAGACTAACC CTCGAG GGTTAGTCTGTTGATTCTGGGTT
shRNABZLF1.2R	5’ AATTCAAAAA - AACCTGGAGACAATTCTACTGTT CTCGAG AACAGTAGAATTGTCTCCAGGTT
shRNABZLF1.3R	5’ AATTCAAAAA - AATGCTTATCAAGCTTATGCAGC CTCGAG GCTGCATAAGCTTGATAAGCATT

#### Generation of Z-HT cell lines and production of virus

Akata, Mutu, AG876 and BL5 BL cells were infected with a retrovirus expressing the BZLF1 protein fused to the hormone domain of the estrogen receptor (Z-HT), constructed as previously described [82], (a gift from Dr. Bill Sugden, University of Wisconsin-Madison), and stable cell lines were selected with at least 1 μg/ml puromycin. To stimulate virus production, ZHT-expressing cell lines were suspended in RPMI complete media with 200 nM of 4-HT (Sigma) at 4 x 10^5^ cells/mL. Cell supernatants were harvested after 4 days of stimulation with 4-HT and concentrated using a Sorvall GSA rotor at 16,000 g for 90 min. Virus was reconstituted at 1:200 initial volume in RPMI. M81 infectious viral particles were produced from a 293 cell line stably infected with M81 virus following by transfecting cells with EBV BZLF1 and BRLF1 expression vectors as previously described [83]. Virus was collected 4 days post-transfection and concentrated as described above.

#### EBV titer determination

EBV-negative Akata cells were infected with serial 10-fold dilutions of GFP-expressing Akata virus (similar to green fluorescent Raji cell titer assay previously described [83]). After 24 h, EBV-infected cells were treated with 20 ng/ml phorbol-12-myristate-3-acetate (PMA; Sigma) and 3 mM sodium butyrate (Sigma), and GFP-expressing cells were counted by fluorescence microscopy 24 h later. The amount of virus required to form one GFP-positive cell was defined as one green Akata infectious unit (GAU). AG876, BL5, Mutu and M81 virus titers were determined by comparing the relative EBNA2 protein expression induced by infection of EBV-negative Akata cells with 5-fold serial dilutions of each virus prep to that induced by serial 5-fold dilution of a known Akata virus titer (Green Akata virus unit (GAU)). Akata, AG876, BL5 and M81 EBV-infected cell lysates were collected at 72 hours after infection, and EBNA2 protein expression was quantitated by immunoblot analysis as described below and assigned a relative GAU titer. Results using an EBNA1 antibody to quantitate virus titers were similar to those obtained using the EBNA2 antibody.

#### EBV infection of humanized NSG mice

Immunodeficient NSG (NOD/LtSz-scid/IL2Rγnull) mice were purchased from Jackson labs (catalogue number: 005557). Commercially available CD34-depleted human cord blood mononuclear cells (AllCells, LLC) were infected with various amounts of each virus as indicated in the results section (2,000 to 20,000 GAUs) *in vitro* for 1.5 hours at 37°C, and a minimum of 10 million cells were injected intraperitoneally (i.p.) into 3–5 weeks-old age matched NSG mice [83]. Mice were kept for maximum time of 42 days after cord blood injection or were euthanized due to tumor symptoms prior to 42 days.

#### IHC analysis of EBV infected tumors and LCLs

Formalin-fixed tumor tissue and organs were examined by multiple techniques to determine if animals had EBV-positive lymphomas, and to assess the viral protein expression pattern. LCLs were suspended in a 1% agar solution and then formalin-fixed (referred to as cell blocks). Analysis for all animals included H&E staining, and IHC staining as previously described [5,6,38,83,84]. For immunohistochemistry, formalin-fixed, paraffin-embedded tissue sections and cell lines were deparaffinized and then examined by IHC as previously described [83,85]. Co-staining of tumor tissue using antibodies against CD20 (B cell marker), CD3 (T cell marker), and EBV latency genes EBNA1 was performed as previously described [4–6,85]. All antibodies used are described in Table 3. Dr. Erik A. Ranheim, a board-certified hematopathologist, performed the pathological analysis of the tumors. IHC quantification was performed as previously described [6]. Briefly, 10 fields of view at 20X magnification were imaged and then the number of Z positive cells were counted. This value was compared to the total number of cell present in the field of view. The data from the 10 fields were averaged. This analysis was performed across 3 different clones from two different type 2 LCLs (AG876 and BL5).

**Table 3:** The list of antibodies used in this study for IHC and immunoblot analysis.

Antibody	Clone	Company	Dilution
CD20 (mouse)	H1	BD Pharmingen	1:500
CD20 (rabbit)	BV11	Abcam, Inc.	1:100
CD3	Polyclonal	DakoCytomation	1:200
IRF4	MUM1p	Santa Cruz Biotechnology, Inc.	1:50
EBNA2	PE2	Abcam, Inc.	1:100
LMP1 (IHC)	CS. 1–4	Abcam, Inc.	1:10
LMP1 (Immunoblot)	CS. 1–4	Abcam, Inc.	1:500
BZLF1	BZ1	Santa Cruz Biotechnology, Inc.	1:100 (IHC) 1:500 (Immunoblot)
gp350	A gift from Dr. Eric Johansen	Polyclonal	1:200
EBNA1 (IHC)	1EBI14	Santa Cruz Biotechnology	1:100
EBNA1 (Immunoblot)	O211	Santa Cruz Biotechnology	1:500
EBNA3A	Polyclonal	Exalpha	1:500
EBNA3B	Polyclonal	Exalpha	1:500
EBNA3C	Polyclonal	Exalpha	1:500
BMRF1 (EA-D)	R3	Millipore	1:1000
BRLF1	Polyclonal		1:2500
p18VCA	Polyclonal	Thermo Fisher	1:1000
Phoshpo-PLCγ2	Polyclonal	Cell Signaling Technology	1:1000
PLCγ2	Polyclonal	Cell Signaling Technology	1:1000
Pan-Calcineurin A	Polyclonal	Cell Signaling Technology	1:1000
MARCKs		Cell Signaling Technology	1:1000
NFATc1	7A6	Santa Cruz Biotechnology, Inc.	1:500
NFATc2	Polyclonal	Cell Signaling Technology	1:1000
HA	16B12	Covance	1:1000
Actin	AC-15	Sigma	1:5000
Lamin B	C-20	Santa Cruz Biotechnology, Inc.	1:200
Tubulin	B-5-1-2	Sigma	1:4000

### Generation of lymphoblastoid cell lines (LCLs)

All LCLs used in these studies were derived from this same donor. To generate LCLs, adult peripheral blood mononuclear cells were isolated by Ficoll gradient, infected with four different virus types (Akata, Mutu, AG876, BL5), and multiple lines derived from each virus type were then used to examine latent and lytic viral protein expression and LCL growth rate. All experiments on LCLs were performed at least 6 to 8 weeks post-infection (using cells infected with each virus type on the same day).

### Immunoblot analysis

Protein samples were harvested from cells in culture as previously described [86], and from frozen primary tumor tissue by homogenizing tissue with the Covaris Cryoprep Pulverizer as previously described [6]. Cell and tissue lysates were harvested in Sumo lysis buffer including protease inhibitors (Roche) and phosphatase inhibitors (Sigma) as described previously [87]. Protein concentration was determined using the Sumo protein assay (Biorad), and proteins were separated in SDS-10% and -15% polyacrylamide gels and transferred onto nitro-cellulose membranes. Membranes were blocked in PBS containing 5% milk, and 0.1% Tween 20 solution. Membranes were then incubated with primary and secondary antibodies listed in Table 3. Image Studio Lite software was used to quantify levels of NFATc2, Z, R, and BMRF1 relative to loading control actin in Fig 11 [20].

### Plasmids and nucleofection

Plasmid DNA was prepared using the Qiagen Maxi prep kit according to the manufacturer’s instructions. Plasmid pcDNA3.1 was obtained from Stratagene. pDUAL CMMP(-) constitutively active NFATc1 and NFATc2 was a gift from Dr. Anjana Rao (La Jolla Institute) through Dr. Alan Attie at the University of Wisconsin. pcDNA3.1-constitutively active CAMKIV was a gift from Dr. Xiang-Jiao Yang (McGill University) [88]. Akata Burkitt lymphoma cells were nucleofected using the Amaxa Nucleofector 2b device (Lonza) and program A-030 (with Buffer V) in 12-well dishes with 1 μg of vector control, constitutively active NFATc1, constitutively active NFATc2, or constitutively active CAMKIV plasmid. The cells were washed with PBS and harvested in SUMO buffer 24 hrs post-nucleofection as described above. Cell extracts were subjected to immunoblot analysis as described above. pHEBo-MT-EBNA2 constructs were constructed as previously described [28]. P3HR1 BL cells were nucleofected using the Amaxa Nucleofector 2b device (Lonza) and program A-030 (with Buffer V) in 12-well dishes with 1 μg of T1 EBNA2 or T2 EBNA2. At 3 days post-nucleofection, cells were treated with 200μg/mL of hygromycin and continually passaged until stable cell line was produced. Stable cells were treated with 10 or 20 μM CdCl2 for 24–48 hours and harvested in SUMO buffer as described above.

### Quantitative PCR analysis of tumor tissue and LCLs

RNA samples were harvested from frozen primary tumor tissue by homogenizing tissue with the Covaris Cryoprep Pulverizer as previously described [6]. The pulverized tissue and LCLs were lysed in Trizol reagent (Invitrogen). RNA was isolated using phenol/chloroform extraction, and total RNA was quantitated using a NanoDrop 2000 Spectrophotometer machine (ThermoFisher). The extracted RNA was then treated with DNase, followed by reverse transcription using random primers and GoScript reverse transcriptase (RT) (Promega). Real-time PCR was performed on the reverse-transcribed cDNA (1 μL) by using iTaq Universal SYBR green mix (Bio-Rad) in a Bio-Rad CFX96 machine for 40 cycles of 15 s at 95°C and 30 s at 60°C [89]. The CFX Maestro software (Biorad) was used to determine Cq values. Cq values were either normalized to the CD20 (MS4A1) value (EBV genes and B-specific genes), total GAPDH, or 18S rRNA. Delta Cq values were determined by calculating 2^ (Normalized Cq) which were then plotted. All primers used in qPCR analysis are listed in Table 4 [36].

**Table 4:** The primers used for qPCR analysis in this study.

Gene	Forward Primer (5’-3’)	Reverse Primer (5’-3’)
18S	GTAACCCGTTGAACCCCATT	CCAT CCAATCGGTAGTAGCG
CD20 (MS4A1)	GCTGGCATCGTTGAGAATGAAT	TGCTGACAGGAGAACTATGTTAGAT
LMP1	TGAGTAGGAGGGTGA	CTATTCCTTTGCTCTCATGC
BARTs	AGAGACCAGGCTGCTAAACA	AACCAGCTTTCCTTTCCGAG
BZLF1	CGCACACGGAAACCACAACAGC	GAAGCGACCTCACGGTAGTG
BcLF1	CCTCAAACCCGTGGATCATAG	GTGGATCAGGCCGTTATTGA

### CRISPR Cas9 knockdown assay

CRISPR experiments were performed using the 4D-Nucleofector system and SF kit (Lonza) as directed by Jiang *et al*. [90]. RNPs were prepared as per the manufacturer’s protocol. The sgRNA (10uM concentration) used against NFATc2 (Table 5) was resuspended in T10E. For each nucleofection reaction (1 well), 2 μl of 10 μM sgRNA (IDT) was mixed with 0.289uL of purified Cas9 (IDT 61pmol/uL) and 0.711uL T10E in 1.5‐ml RNase‐free microcentrifuge tubes and incubated for 5 min at room temperature prior to nucleofection. 1 million cells were used per well. Experimental conditions were Cas9 alone and Cas9 +NFATc2. Cells were nucleofected with the DN-100 program, incubated 72 hr in RPMI/10% fetal calf serum, then harvested in SUMO lysis buffer.

**Table 5:** The sgRNA used in this study.

Gene	sgRNA
NFATc2	mGmAmUCCCACAAGGCGAGUCCGGUUUUAGAGCUAGAAAUAGCAAGUUAAAAUAAGGCUAGUCCGUUAUCAACUUGAAAAAGUGGCACCGAGUCGGUGCmUmUmUU

### Microscopy and image analysis

Cells were stained with BioTracker 609 Red Ca2+ AM Dye (Millipore Sigma, Burlington, MA, USA) and NucBlue LiveCell Stain ReadyProbes (Life Technologies, Carlsbad, CA, USA) following the manufacturer’s protocol prior to imaging. 2.5x10^5 cells were plated into 8-well chamber μ-slides (IBIDI, Madison, WI, USA) and imaging was performed shortly after plating using a Nikon Ti-Eclipse inverted wide-field microscope (Nikon Corp., Tokyo, JPN) and a 20x Plan Apo objective lens (NA, 0.75) as previously described [91]. Image acquisition for each well was performed using an Orca-Flash4.0 Digital Camera C11440 (Hamamatsu, JPN) and Nikon NIS Elements software (v4.13.04). Each image was collected using differential interference contrast (DIC) and the following fluorescence excitation/emission (nanometer ranges) filter sets: 325 to 375/435 to 485 (NucBlue) and 565 to 590/590 to 650 (BioTracker 609 Red Ca2+ AM Dye). For each condition, four fields of view (FOV) were acquired with a 20x Plan Apo objective lens and 10% light intensity. All images were processed and analyzed using FIJI/ImageJ2 software [92]. Each FOV was processed in FIJI as follows: background was subtracted using a rolling ball radius of 50 pixels prior to measurement of integrated density (area x mean gray value) for each channel (NucBlue, Ca2+ Dye) imaged. For each condition, cells within each FOV were used to calculate the relative fluorescence intensity (RFU) through measurement of the average integrated density of Ca2+ positive cells normalized to total number of cells (NucBlue—nuclei stain).

### Statistics

All bar graphs were constructed in Microsoft Excel and standard error was used for error bars in graphs. Fisher Exact and Wilcoxon Rank Sum tests were performed using MSTAT statistical software (https://mcardle.wisc.edu/mstat/) to determine if there was a difference in tumor incidence between virus strains. A p-value of < .05 was considered significant in all tests used.

## Supporting information

S1 Fig BL5-infected lymphomas have elevated lytic infection compared to Mutu-infected lymphomas and have no detectable T cell infection. **A)** IHC analysis using antibodies against EBNA2 (EBV latency protein), BZLF1 (Z) (immediate-early lytic protein), and gp350 (late lytic protein) was performed as indicated. Arrows indicate gp350-positive cells. **B)** IHC co-staining analysis was performed on T1 and T2 EBV-induced lymphomas using antibodies that recognize EBNA1 (EBV latency gene), CD20 (B cell marker) and CD3 (T cell marker) as indicated. Co-staining cells are indicated with the arrows.
(TIF)

S2 Fig Reduction of Z in AG876 LCLs to comparable levels observed in Mutu LCLs does not rescue cell growth. **A)** T1 EBV-infected (Mutu) or T2 EBV-infected (AG876) LCLs stably infected with control shRNA or shRNAs targeting Z were diluted to 1x10^5 cells and counted 72 hours later. The fold increase in cell number was determined by comparing cell counts at 72 hours to initial cell number. Experiment was performed in triplicate. **B)** Immunoblot analysis of AG876 or Mutu LCLs infected with either control shRNA or shRNA targeting Z using antibodies against Z and actin.
(TIF)

S3 Fig Lytic infection depends upon NFAT, PLCγ, BTK, and PKC activityin AG876 LCLs. A) AG876 LCLs were treated with inhibitors that target various components of the BCR pathway, including the PLCγ inhibitor, U73133, the NFAT inhibitor, cyclosporin A, the BTK inhibitor, Ibrutinib, and the PKC inhibitor, PKC412. Extracts were harvested 48 hours and immunoblot analysis was performed using antibodies against the EBV Z protein and actin as indicated. B) Immunoblot analysis of T1 and T2 LCLs using antibodies against LMP2A and actin.
(TIF)

S4 Fig Quantification of NFATc2 knockdown and reduction of lytic gene expression. Densiometry analysis on immunoblots was performed to quantitate knockdown of NFATc2 by sgRNA (Fig 11B) and shRNA (Fig 11C). Fold change is shown after normatlization to Actin. Densiometry analsysis was also performed to quantitate knockdown of Z, R, and BMRF1 lytic EBV gene expression when NFATc2 was knocked down in LCLs treated with sgRNA (Fig 11B) and shRNA (Fig 11C).
(TIF)
