# Supplementary figures and images for: B cells infected with Type 2 Epstein-Barr virus (EBV) have increased NFATc1/NFATc2 activity and enhanced lytic gene expression in comparison to Type 1 EBV infection
Source: PLoS Pathog. 2020 Feb 14;16(2):e1008365. doi: 10.1371/journal.ppat.1008365 (PMC7046292; doi:10.1371/journal.ppat.1008365)

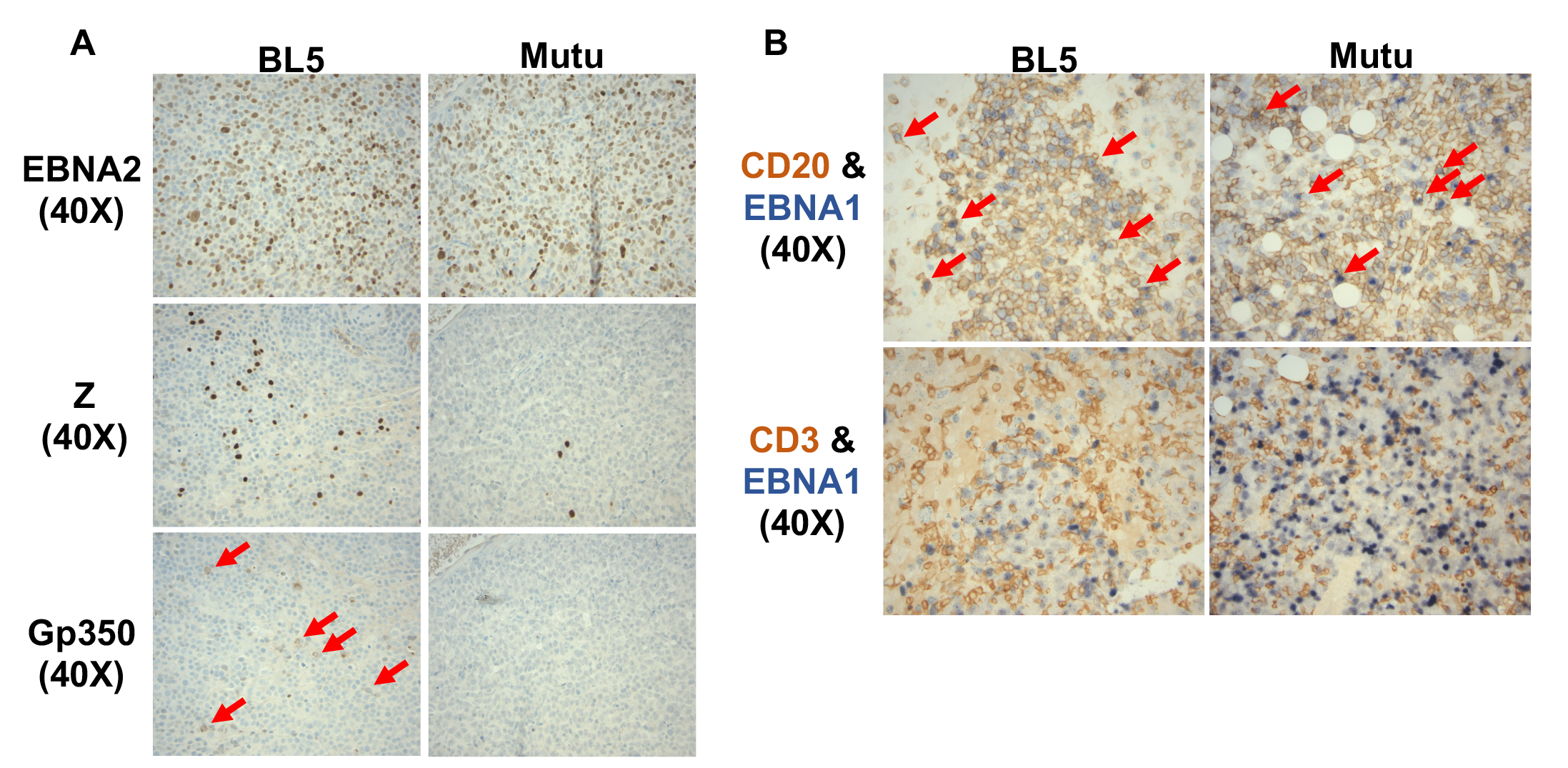

Supplement: S1 Fig — A) IHC analysis using antibodies against EBNA2 (EBV latency protein), BZLF1 (Z) (immediate-early lytic protein), and gp350 (late lytic protein) was performed as indicated. Arrows indicate gp350-positive cells. B) IHC co-staining analysis was performed on T1 and T2 EBV-induced lymphomas using antibodies that recognize EBNA1 (EBV latency gene), CD20 (B cell marker) and CD3 (T cell marker) as indicated. Co-staining cells are indicated with the arrows. (TIF) [file ppat.1008365.s001.tif]

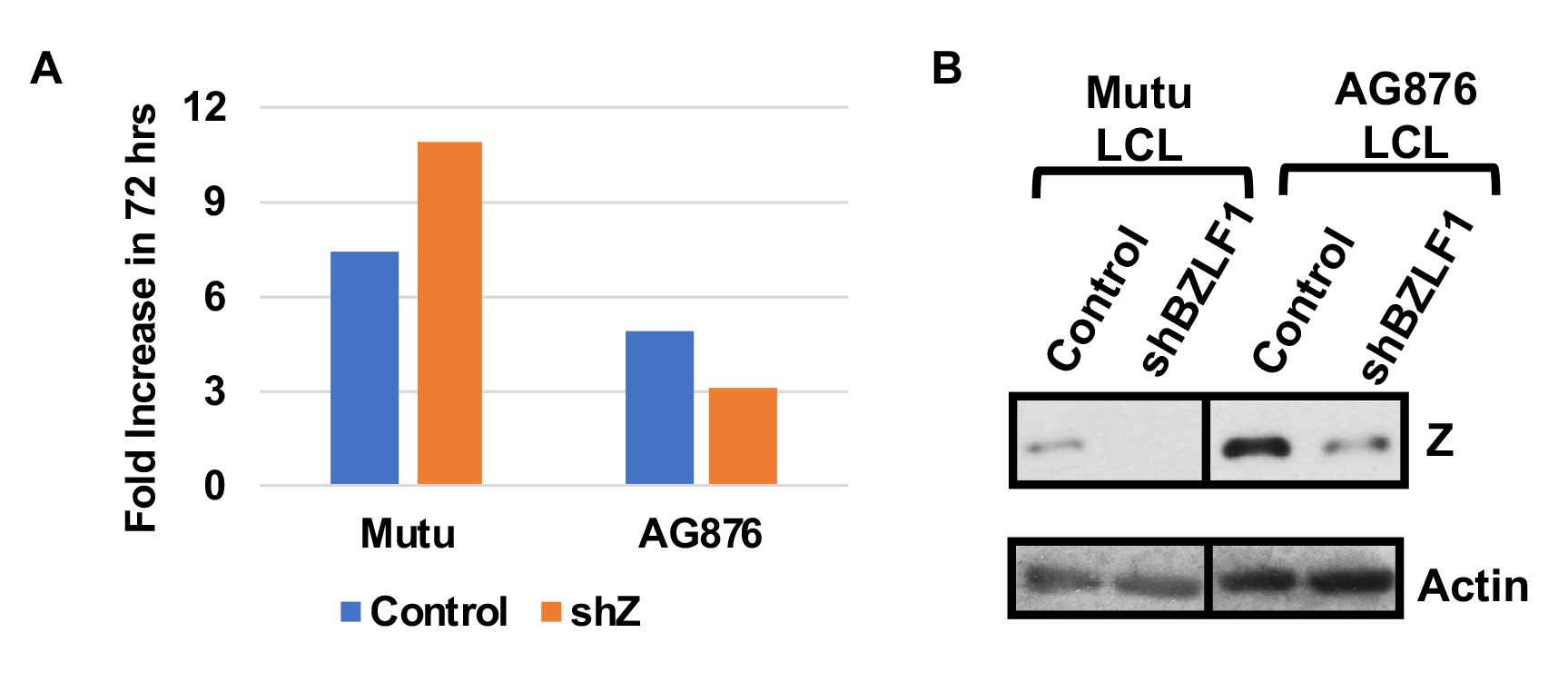

Supplement: S2 Fig — A) T1 EBV-infected (Mutu) or T2 EBV-infected (AG876) LCLs stably infected with control shRNA or shRNAs targeting Z were diluted to 1x10^5 cells and counted 72 hours later. The fold increase in cell number was determined by comparing cell counts at 72 hours to initial cell number. Experiment was performed in triplicate. B) Immunoblot analysis of AG876 or Mutu LCLs infected with either control shRNA or shRNA targeting Z using antibodies against Z and actin. (TIF) [file ppat.1008365.s002.tif]

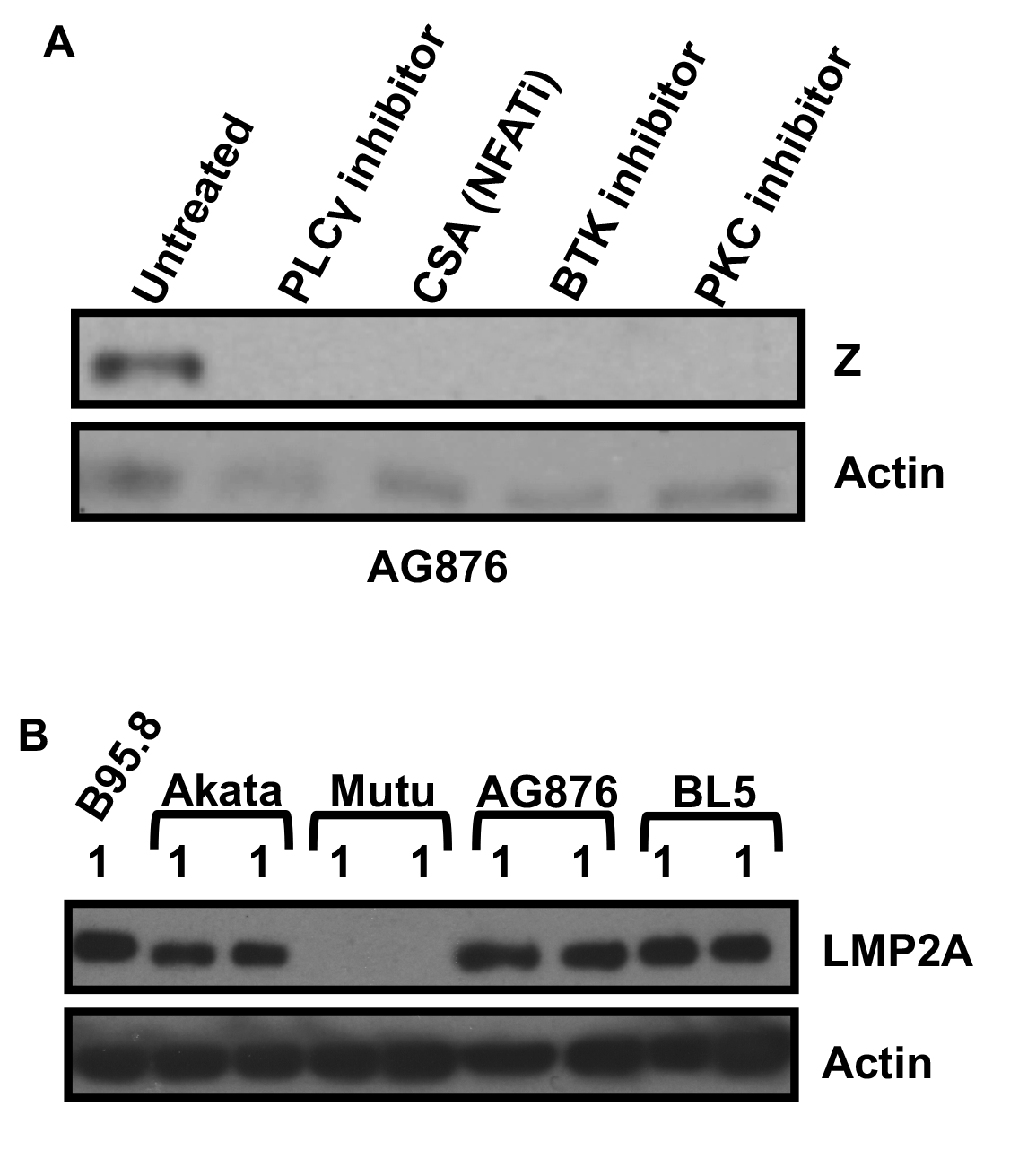

Supplement: S3 Fig — A) AG876 LCLs were treated with inhibitors that target various components of the BCR pathway, including the PLCγ inhibitor, U73133, the NFAT inhibitor, cyclosporin A, the BTK inhibitor, Ibrutinib, and the PKC inhibitor, PKC412. Extracts were harvested 48 hours and immunoblot analysis was performed using antibodies against the EBV Z protein and actin as indicated. B) Immunoblot analysis of T1 and T2 LCLs using antibodies against LMP2A and actin. (TIF) [file ppat.1008365.s003.tif]

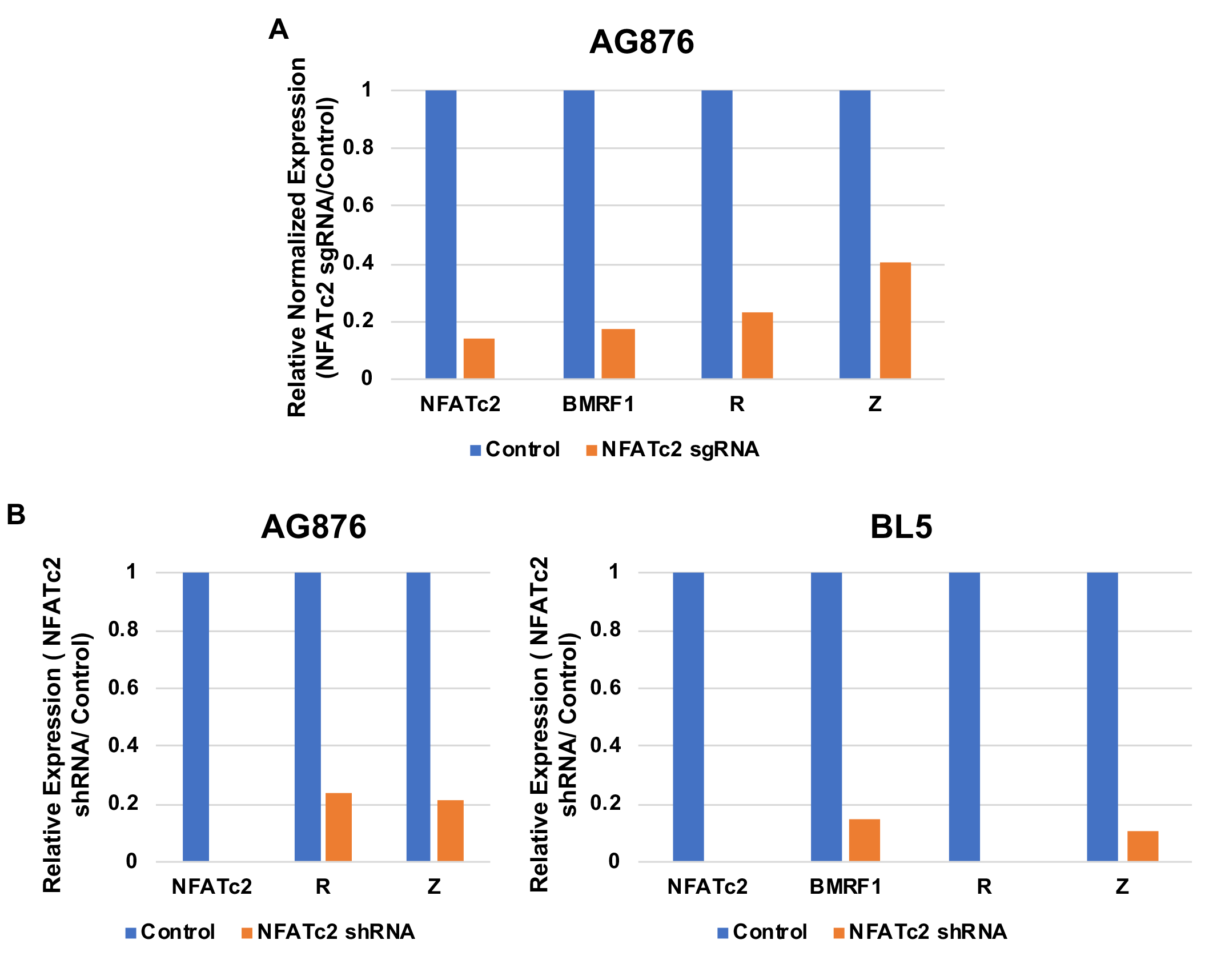

Supplement: S4 Fig — Densiometry analysis on immunoblots was performed to quantitate knockdown of NFATc2 by sgRNA (Fig 11B) and shRNA (Fig 11C). Fold change is shown after normatlization to Actin. Densiometry analsysis was also performed to quantitate knockdown of Z, R, and BMRF1 lytic EBV gene expression when NFATc2 was knocked down in LCLs treated with sgRNA (Fig 11B) and shRNA (Fig 11C). (TIF) [file ppat.1008365.s004.tif]
